# Exploring the Relationship Between Deficits in Social Cognition and Neurodegenerative Dementia: A Systematic Review

**DOI:** 10.3389/fnagi.2022.778093

**Published:** 2022-04-27

**Authors:** Esther Setién-Suero, Nancy Murillo-García, Manuel Sevilla-Ramos, Georgelina Abreu-Fernández, Ana Pozueta, Rosa Ayesa-Arriola

**Affiliations:** ^1^Department of Psychiatry, School of Medicine, University of Cantabria, University Hospital Marqués de Valdecilla, Santander, Spain; ^2^IDIVAL, Valdecilla Biomedical Research Institute, Santander, Spain; ^3^Department of Psychology, Faculty of Health Sciences, University of Deusto, Bilbao, Spain; ^4^Neurology Service and Centro de Investigación Biomédica en Red sobre Enfermedades Neurodegenerativas (CIBERNED), Madrid, Spain; ^5^CIBERSAM, Biomedical Research Network on Mental Health Area, Madrid, Spain

**Keywords:** neurodegenerative disease, dementia, social cognition, theory of mind, emotional processing, social perception, attribution bias

## Abstract

**Background:**

Neurodegenerative diseases might affect social cognition in various ways depending on their components (theory of mind, emotional processing, attribution bias, and social perception) and the subtype of dementia they cause. This review aims to explore this difference in cognitive function among individuals with different aetiologies of dementia.

**Methods:**

The following databases were explored: MEDLINE via PubMed, Cochrane Library, Lilacs, Web of Science, and PsycINFO. We selected studies examining social cognition in individuals with neurodegenerative diseases in which dementia was the primary symptom that was studied. The neurodegenerative diseases included Alzheimer's disease, Lewy body disease and frontotemporal lobar degeneration. The search yielded 2,803 articles.

**Results:**

One hundred twenty-two articles were included in the present review. The summarised results indicate that people with neurodegenerative diseases indeed have deficits in social cognitive performance. Both in populations with Alzheimer's disease and in populations with frontotemporal dementia, we found that emotional processing was strongly affected. However, although theory of mind impairment could also be observed in the initial stages of frontotemporal dementia, in Alzheimer's disease it was only appreciated when performing highly complex task or in advanced stages of the disease.

**Conclusions:**

Each type of dementia has a differential profile of social cognition deterioration. This review could provide a useful reference for clinicians to improve detection and diagnosis, which would undoubtedly guarantee better interventions.

**Systematic Review Registration:**

https://www.crd.york.ac.uk/prospero/display_record.php?ID=CRD42020152562, PROSPERO, identifier: CRD42020152562.

## Introduction

In the framework of cognitive neuroscience, social cognition is considered an independent component of cognitive functioning (American Psychiatric Association, 2013; Happe and Conway, 2016; Duclos et al., 2018a; Garcia et al., 2018). It is broadly defined as the set of mental operations that underlie social interactions and encompasses multiple processes, including perception of self and other people, interpretation of the behaviours and intentions of others, and knowledge of interpersonal and social norms (Fiske, 1991; Beer and Ochsner, 2006; Pinkham, 2014). To date, a consensus has not been reached on which domains compose social cognition, but the most extensively studied components are theory of mind (ToM), emotional processing, social perception, and attribution bias (Hoertnagl and Hofer, 2014; Pinkham, 2014; Healey et al., 2016; Garcia et al., 2018). ToM, also known as mentalizing, refers to the ability to infer the mental states of other individuals and has cognitive and affective subcomponents (Mar, 2011; Elamin et al., 2012; Happe and Conway, 2016). Cognitive ToM involves the recognition that the beliefs of other people might be different from one's own beliefs, and affective ToM involves the inference of one's emotional state (Mar, 2011; Elamin et al., 2012). Emotional processing is described as perceiving and using emotional information adaptively and includes the skills needed to recognise, manage, and regulate emotions (Green and Horan, 2010; Pinkham, 2014). Social perception is the ability to decode and interpret social cues, such as identifying social roles, societal rules, and social context (Green and Horan, 2010; Pinkham, 2014). Attribution bias refers to how the individual typically explains the causes of positive and negative social events (Green and Horan, 2010). Evidence suggests that these processes are associated with the activation of a complex neuronal network involving white matter regions (Wang and Olson, 2018) and cortical regions, such as the prefrontal cortex, the paracingulate cortex, the temporoparietal junctions, the amygdala, and the hippocampus (Mar, 2011; Skuse and Gallagher, 2011; Christidi et al., 2018; Garcia et al., 2018). Consequently, neurodegenerative diseases that disrupt this neurobiological system might cause dysfunctions in social cognition that produce abnormal interpersonal behaviour (Duclos et al., 2018a).

The most common neurodegenerative diseases are Alzheimer's disease (AD), vascular disease, Lewy body disease (DLB), and frontotemporal lobar degeneration (FTD). These pathologies primarily affect older people and lead to a neurobehavioural syndrome known as dementia (American Psychiatric Association, 2013; World Health Organization WHO, 2019) or major neurocognitive disorder (American Psychiatric Association, 2013) that interferes with independence in daily life activities (Chertkow et al., 2013; Baez et al., 2014). Approximately 50 million people have dementia worldwide, and this number is projected to reach 82 million by 2030. Current criteria for its diagnosis recognise substantial cognitive decline that affects one or more domains of cognitive functioning, including attention, executive function, learning and memory, language and perceptual-motor abilities, and social cognition (American Psychiatric Association, 2013; Christidi et al., 2018; Duclos et al., 2018a). Therefore, studies of the deterioration of social cognition in individuals with dementia are necessary, as they can improve our understanding of the functioning of this cognitive domain and its neuroanatomical basis.

A growing body of literature suggests that deficiencies in social cognition vary according to its subcomponents and the aetiology underlying the dementia subtype (Christidi et al., 2018; Duclos et al., 2018a; Multani et al., 2019) Loss of empathy (Baez et al., 2016a), ToM alterations (Strikwerda-Brown et al., 2019), and attribution bias (Baez et al., 2014) have been reported in individuals with behavioural variant of FTD (bvFTD). Similarly, people with primary progressive aphasia (another subtype of FTD), in its semantic variant (svPPA), have shown a loss of empathy at the earliest phases of the illness (Duval et al., 2012). A different pattern of decline has been described in individuals with AD because patients tend to retain some social skills during early stages of the disease, which progressively deteriorate during later stages (Strikwerda-Brown et al., 2019). The characteristic neuropsychological profile of each neurodegenerative disease is potentially associated with this diversity by influencing specific patterns of social and emotional impairments (Levy and Chelune, 2007; Hugo and Ganguli, 2014). Some reviews have been performed to clarify the various profiles of deterioration of social cognition among people with different subtypes of dementia. However, they have some weaknesses, such as focusing on a single component of social cognition (Strikwerda-Brown et al., 2019), lacking a systematic bibliographic search, not describing the methodology (Christidi et al., 2018; Duclos et al., 2018a; Strikwerda-Brown et al., 2019) or being outdated (Elamin et al., 2012).

The present systematic review aimed to overcome previous limitations by describing the current evidence for deficits social cognition in individuals with dementia caused by neurodegenerative diseases. The main objective was to analyse its components (ToM, emotional processing, social perception, and attribution bias) across the different aetiologies of dementia. Studies published regarding social cognition in dementia due to neurodegenerative diseases were compiled and organised. All neurodegenerative diseases in which dementia was the main symptom, corresponding to those formerly known as primary degenerative dementias, were included (Reisberg et al., 1982; Grassetto et al., 2014; Onandia-Hinchado and Diaz-Orueta, 2020). In contrast, neurodegenerative diseases whose core symptoms are different from cognitive deficits were excluded.

## Methods

This review is being reported in accordance with the reporting guidance provided in the Preferred Reporting Items for Systematic Reviews and Meta-Analysis (PRISMA) guidelines (Moher et al., 2010). A protocol guided this review (doi: 10.21203/rs.3.rs-28796/v1), and this review was registered in the International Prospective Registry of Systematic Reviews (PROSPERO) with registration number CRD42020152562.

### Eligibility Criteria

All studies that met the following inclusion criteria were included in the review: (a) studies with adult populations that present some type of neurodegenerative disease in which dementia is the main symptom (AD, FTD, and DLB), and these patients were evaluated by an experienced professional according to standardised diagnostic criteria; (b) studies that evaluated any dimension of social cognition (ToM, emotional processing, social perception or attribution bias); (c) observational studies, including cross-sectional, longitudinal, case-control, and cohort studies; (d) studies published in English or Spanish; and (e) studies published between October 2009 and April 2021.

Notably, all FTD types were included. FTD covers bvFTD and primary progressive aphasia (PPA) along with its three variants: (i) the non-fluent variant of PPA (nfvPPA), (ii) semantic variant of PPA (svPPA) (also called semantic dementia), and (iii) logopenic variant of PPA (lvPPA) (Gorno-Tempini et al., 2011).

The following studies were excluded from the review: (a) studies on populations with degenerative disorders in which dementia syndrome was not the main manifestation of the disorder (e.g., vascular disease, dementia in Parkinson's disease, dementia in Huntington's disease, and dementia in Wilson's disease); (b) reviews or meta-analyses; (c) single case studies, comments, books, conference papers, letters, editorials, theses and all studies not peer-reviewed (grey literature).

### Search Strategies and Data Sources

An expert librarian from the Marqués de Valdecilla University Hospital (Santander, Spain) was consulted to establish the search strategy necessary to identify all relevant articles. A systematic search of the following databases was performed: MEDLINE database via PubMed, Cochrane Library, Lilacs, Web of Science (WoS), and PsycINFO. Appropriate search terms were used, namely, MeSH terms, and when these terms were not available, free text was considered using keywords related to dementia and social cognition. In addition, the references of identified studies were also searched to identify additional articles. All studies published in English or Spanish between October 2009 and April 2021 were considered. The detailed search strategy in PubMed was “Dementia” [MeSH] AND (“Social Cognition” OR “Theory of Mind” [MeSH] OR “Social Perception” [MeSH] OR “Emotional Intelligence” [MeSH] OR “Social Knowledge” OR “Attributional Style” OR “Attribution Bias”). The detailed search strategy is shown in Additional File 1 (Supplementary Material).

### Study Selection

All the references retrieved from the different databases in response to the search criteria were imported into the EndNote (software) program (Clarivate Analytics, Philadelphia, PA, USA), which eliminated duplicate citations. The work team, which was composed of four reviewers (ESS, NMG, MSR, and GAF), independently evaluated each of the selected titles and abstracts according to the eligibility criteria. The full texts of potentially relevant articles were retrieved and reviewed again. Each study was evaluated by at least two reviewers. The final decision regarding the inclusion of studies was based on a thorough review of the full articles by two reviewers. In case of discrepancies, these studies were evaluated by the entire team, as well as a senior researcher (RAA). The result of the selection process is reported in a PRISMA flow diagram (Moher et al., 2009).

### Assessment of the Risk of Bias of Primary Studies

We used the Joanna Briggs Institute (JBI) Critical Evaluation Checklists to assess the risk of bias in the different studies (Moola et al., 2017). JBI tools are useful for identifying the strengths and weaknesses of a research article to assess the utility and validity of research findings in a systematic manner. Because the type and propensity for bias of the different studies varies depending on the design of each study, the following checklists were used: (a) JBI critical appraisal checklist for analytical cross-sectional studies, (b) JBI critical appraisal checklist for case-control studies, and (c) JBI critical appraisal checklist for cohort studies. Each of these lists has specific questions addressing bias, confounding variables, statistical analyses, methodological validity, and outcome reliability.

### Data Extraction

Data were extracted into a registry predesigned *ad-hoc*. All members of the review team participated in the design of the registry. The following information was extracted from all included studies: author, year of publication, N, population (type of dementia), type of study, mean age, domain of social cognition evaluated, tool employed to assess social cognition, summary of results, follow-up period (if applicable), and data from the control group (if applicable). The data extraction task was performed by various team members, and each article was independently reviewed by at least two reviewers.

During the data extraction process, the consensus of the entire team was used to resolve discrepancies before reaching a final decision.

### Data Synthesis

Attending the type of dementia studied, the results were grouped into several sections. Specifically, the results were reported in eight sections: (1) social cognition in AD; (2) social cognition in bvFTD; (3) social cognition in PPA; (4) social cognition in AD compared with bvFTD; (5) social cognition in AD compared with PPA; (6) social cognition in bvFTD compared with PPA; (7) social cognition in unspecific FTD; and (8) social cognition in DLB. Within these sections, an attempt to synthesise the results with respect to the different domains of social cognition was made. Results were organised following the chronological model described in the introduction, which begins with the perception of oneself and other people, and continues with the interpretation of behaviours and intentions and ends with the knowledge of the rules and their regulation (Fiske, 1991; Beer and Ochsner, 2006; Pinkham, 2014); that is: ToM, emotional processing, social perception, and attribution bias.

## Results

**Figure 1:**
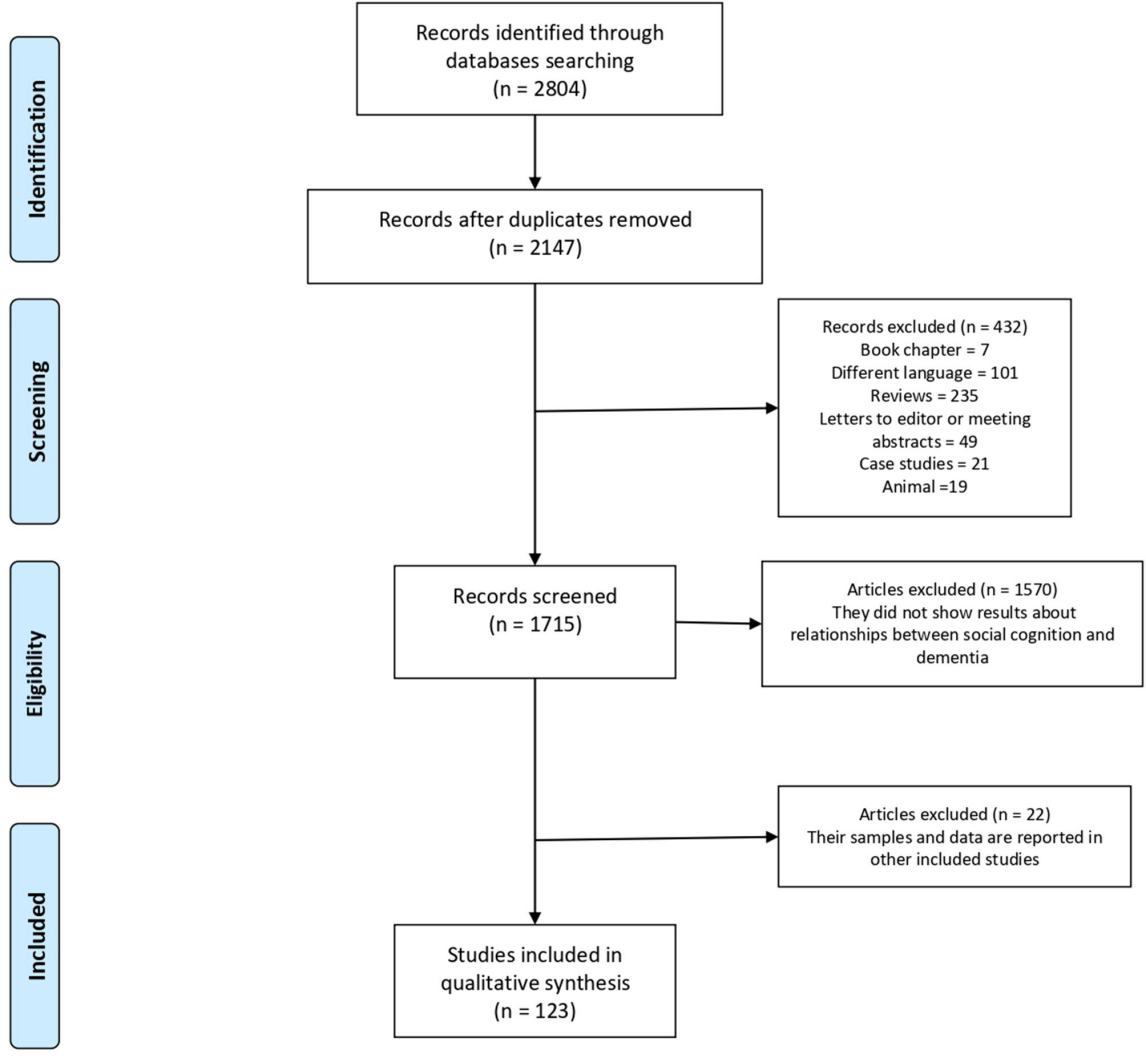
Flow diagram selection of the study process.

Of the 2804 identified articles, 2147 were selected based on search criteria after duplicates were removed. Then, 1715 articles were chosen as potentially relevant after discarding 432 studies for different reasons (7 book chapters, 101 articles published in different languages, 235 reviews, 49 letters to the editor or meeting abstracts, 21 case studies and 19 animal studies). Among those remaining articles, another 1570 studies were excluded after reading the abstracts, attending they did not explore any domain of social cognition in any of the neurodegenerative dementias under study. Of the 145 articles that met the inclusion criteria, 22 studies were excluded because they contained overlapping data. In those cases when the data from the same sample were presented in several studies, the first publication was considered. Finally, 123 articles qualified for the present review (Figure 1). Data from all included studies are summarized in Tables 1–8.

**Table 1:** Summary of studies on social cognition in Alzheimer's disease.

**References**	**Design**	**Patients (*N*)**	**Mean age of patients (S.D)**	**HC (*N*)**	**Mean age of HC (S.D)**	**SC domains**	**Assessment tools**	**Results**	**JBI**
García-Rodríguez et al. (2009)	C-S	16	77.5 (4.34)	16	73.25 (5.37)	EP	FACS	No significant results	M
Guaita et al. (2009)	C-S	79	80.65 (8.39)	64	76.05 (7.10)	EP	*Ad-hoc* test	No significant results	M
Henry et al. (2009)	C-S	20	80.5 (5.93)	20	81.8 (4.23)	ToM	RMET	No significant results	M
Youmans and Bourgeois (2010)	C-S	10	82 (6.15)	10	78 (6.11)	ToM	FBT	Patients impaired in ToM	G
Castelli et al. (2011)	C-S	16	70.5 (5.71)	16	71.38 (3.65)	ToM	FBT, RMET	Patients impaired in second-order FBT	M
Yamaguchi et al. (2012)	C-S	Mi: 36; Mo: 14	74.4 (5.0); 79.2 (5.8)	26	77.1 (6.5)	ToM	PT	ToM impairment progresses with disease	G
Choong and Doody (2013)	C-S	Mi:7; Mo:9	65–87	11	65–87	ToM	SSTMT, CJT	No significant results	M
Laisney et al. (2013)	C-S	16	78.1 (2.6)	15	76.4 (3.2)	ToM	FBT, RMET, PJ	No significant results	G
Maki et al. (2013a)	C-S	30	78 (7.2)	Young: 31 Normal: 104	19.3 (1.4) 72.1 (4.2)	ToM	MSST	ToM impairment progresses with disease	L
Maki et al. (2013b)	C-S	12	81.1 (9.2)	Young: 25 Normal: 17	18.9 (1.1) 76.8 (3.5)	ToM	*Ad-hoc* test	Patients impaired in second-order FBT	M
Kumfor et al. (2014b)	C-S	18	65.7 (7.0)	22	65 (5.7)	EP	FERT, TASIT, Ekman 60 Task	Patients impaired in EP	M
El Haj et al. (2015)	C-S	28	74.38 (6.63)	30	70.27 (9.05)	ToM	RMET, FBT	Patients impaired in second-order FBT	M
Insch et al. (2015)	C-S	15	75.25 (6.46)	15	73.13 (5.21)	EP	*Ad-hoc* test	Patients impaired in EP	M
Moyse et al. (2015)	C-S	45	75.46 (5.64)	45	74.69 (5.68)	AB	*Ad-hoc* test	Patients impaired in AB	L
Torres et al. (2015)	Lo	30	77.23 (7.21)	N/A	N/A	EP	FACES	Significantly worse performance over time	M
Fliss et al. (2016)	C-S	42	78.5 (8.45)	23	77.9 (9.9)	ToM	FBT, EFT, PJ	Patients impaired in second-order FBT	M
Moreau et al. (2016)	C-S	20	77.9 (5.5)	20	75.7 (6.1)	ToM	FBT, TRCT	Patients impaired in second-order FBT	M
Daley et al. (2018)	C-S	28	79.9 (7.1)	30	77.9 (8.4)	EP	ACSSP	Patients impaired in EP	M
Antonio Garcia-Casal et al. (2017)	Lo	36	77.48 (5.20)	N/A	N/A	EP	Affect-GRADIOR	EP improved after training	M
Insch et al. (2017)	C-S	24	74.56 (5.70)	24	74.00 (5.43)	SP	*Ad-hoc* test	Patients impaired in SP	M
Poveda et al. (2017)	C-S	27	78.9 (4.83)	27	78 (6.22)	SP	TASIT	Patients impaired in SP	M
Sava et al. (2017, 2019)	C-S	17	78.82 (3.24)	Young: 25 Old: 21	19.84 (2.03) 74.33 (8.47)	EP	Sss*ad-hoc* test	Patients impaired in EP	M
Simm et al. (2017)	C-S	49	60 (N/A)	26	60 (N/A)	SP	RSFS	Patients impaired in SP	M
Duclos et al. (2018b)	C-S	20	79.4 (5.1),	Young: 20 Old: 20	24.6 (2.3) 77.3 (5.9)	ToM	*Ad-hoc* test	Patients impaired in second-order FBT	G
Perri et al. (2018)	C-S	20	76.83 (5.2)	20	73.50 (6.91)	ToM	FBT	Patients impaired in second-order FBT	M
Takenoshita et al. (2018)	C-S	116	79.2 (6.7)	35	73.2 (5.1)	ToM	Sally-Anne test	Patients impaired in second-order FBT	M
Yamaguchi et al. (2018)	C-S	Mi: 34; Mo: 17	79.5 (6.1); 82.4 (5.1)	45	73.2 (5.0)	ToM	*Ad-hoc* test	ToM impairment progresses with disease	G
Antonio Garcia-Casal et al. (2019)	C-S	84	78.27 (5.8)1	69	73.14 (6.28)	EP	Affect-GRADIOR	Patients impaired in EP	G
Lozachmeur et al. (2019)	C-S	30	73.18 (7.18)	33	71.03 (7.09)	ToM	Cartoons FBT	Patients impaired in second-order FBT	M
Arroyo-Anlló et al. (2019)	C-S	Mi: 13; Mo: 17	74.8 (3.5); 76.1 (2.9)	30	75.9 (1.3)	EP	*Ad-hoc* test	Patients impaired in EP	M
Dourado et al. (2019)	C-S	Mi: 29; Mo; 23	77.9 (7.5); 80.2 (8.1)	N/A	N/A	EP	FACES	Patients impaired in EP	G
El Haj et al. (2019)	C-S	26	73.08 (6.66)	28	70.64 (8.99)	ToM	FBT	Patients impaired in second-order FBT	G
Chainay and Gaubert (2020)	C-S	28	74.4 (7.9)	33	72.6 (6.1)	ToM	MSFDE, FPT	Patients impaired in ToM	M
Hayashi and Terada (2021)	C-S	Mi: 52; Mo: 44	76.1 (8.2); 78.1 (6.6)	32	74.6 (9.5)	EP	FERT	Patients impaired in EP	M

*AB, Attribution Bias; ACSSP, Advanced Clinical Solutions Social Perception subtest; CJT, Cartoon joke tasks; C-S, Cross-sectional; EFT, Eyes/Faces Test; EP, Emotional processing; FACES, Facial Expression Recognition Ability; FACS, Facial Action Coding System; FBT, False belief task; FERT, Facial Emotion Recognition Task; FPT, Faux pas test; G, Good; HC, Healthy Controls; JBI, Joanna Briggs Institute Critical Evaluation Checklists; L, Low; Lo, Longitudinal; Mi, Middle; Mo/M, Moderate; MSFDE, Montreal Set of Facial Displays of Emotion; MSST, Metaphoric and Sarcastic Scenario Test; N/A, not available; PJ, Preference Judgement Task; PT, Pitfall Task; RMET, Reading the Mind in the Eyes Test; RSFS, Reflective Self-Function Scale; SC, Social Cognition; SP, Social Perception; SSTMT, Short storey theory of mind task; TASIT, The Awareness of Social Inference Test; ToM, Theory of Mind; TRCT, The Referential Communication Task*.

**Table 2:** Summary of studies on social cognition in the behavioural variant frontotemporal dementia.

**References**	**Design**	**Patients (*N*)**	**Mean age of patients (S.D)**	**HC (N)**	**Mean age of HC (S.D)**	**SC domains**	**Assessment tools**	**Results**	**JBI**
Torralva et al. (2009)	C-S	35	G1: 65 (7.4), G2: 69.1(5.7)	14	65.5 (6.5)	ToM	RMET, FPT	Patients impaired in ToM	G
Grossman et al. (2010)	C-S	19	62.38 (12.30)	19	72.55 (6.85)	ToM	*Ad-hoc* test	Patients impaired in ToM	M
Baez et al. (2014)	C-S	37	66.0 (7.43)	30	55 (8.64)	ToM, EP	TASIT, RMET, EPT, SNQ	Patients impaired in EP	G
Cerami et al. (2014)	C-S	18	63.36 (7.47)	36	62.83 (7.95)	ToM, EP, AB	*Ad-hoc* test	Patients impaired in AB	M
Savage et al. (2014)	C-S	54	62.5 (9.9)	30	63.4 (4.2)	EP	TASIT, EDT	Patients impaired in EP	G
Cerami et al. (2015)	C-S	17	67.88 (9.92)	N/A	N/A	EP	E60, SET, IRI	Patients impaired in EP	G
Custodio et al. (2015)	C-S	28	65.15 (2.95)	20	66.4 (3.9)	ToM	FBT, RMET	Patients impaired in ToM	G
Oliver et al. (2015)	C-S	24	64.7 (7.9)	24	65 (8.5)	EP	IRI; MET	Patients impaired in EP	G
Torralva et al. (2015)	C-S	14	69.9 (8.5)	18	64.5 (6.4)	ToM	RMET, FPT	Patients impaired in ToM	G
Van den Stock et al. (2015)	C-S	20	65.7 (8.7)	22	66.6 (6.1)	EP	*Ad-hoc* test	Patients impaired in EP	G
Baez et al. (2016b)	C-S	21	63.80 (7.33)	19	60.42 (6.77)	AB	*Ad-hoc* test	Patients impaired in AB	G
Jastorff et al. (2016)	C-S	14	67.2 (8.4)	19	66.5 (6.3)	EP	*Ad-hoc* test	Patients impaired in EP	G
Sedeno et al. (2016)	C-S	14	66.42 (6.83)	12	62.58 (6.30)	ToM	TASIT; RMET	Patients impaired in ToM	G
Tabernero and Politis (2016)	C-S	26	68.0 (7.0)	23	68.0 (7.0)	EP, ToM	E60, FPT, RMET	Patients impaired in all domains	M
Tabernero et al. (2017)	C-S	26	67.42 (6.41)	30	69.97 (8.2)	EP, ToM	RMET, FPT, IGT	Patients impaired in all domains	M
Van den Stock et al. (2017)	C-S	13	66.6 (7.22)	19	66.5 (6.28)	EP, SP, AB	*Ad-hoc* test, RMET	Patients impaired in all domains	G
Gossink et al. (2018)	Lo	22	62.8 (6.7)	N/A	N/A	EP, ToM	E60, FPT	bvFTD more impaired than other ND	M
Reus et al. (2018)	Lo	34	63.2 (6.7)	N/A	N/A	EP, ToM	E60, FPT	No change over time in EP or ToM	G
Schroeter et al. (2018)	C-S	86	63.9 (9.6)	43	66.1 (10.1)	ToM	RMET	Patients impaired in ToM	G
Giovagnoli et al. (2019)	C-S	14	56.79 (14.92)	14	56.57 (12.05)	ToM	FPT	Patients impaired in ToM	G
Van den Stock et al. (2019)	C-S	15	67.3 (6.65)	19	66.6 (6.45)	ToM	FH	Patients impaired in ToM	G
Kawano et al. (2020)	C-S	23	60 (7)	30	59 (8)	EP	Ekman	Patients impaired in EP	M
Lillo et al. (2020)	C-S	20	61.5 (6.3)	21	59.2 (8.8)	ToM, EP	Mini-SEA	Patients impaired in EP and ToM	M

*AB, Attribution Bias; ADFES, Amsterdam Dynamic Facial Expression Set; CATS, Affect Matching subtest of the Comprehensive Affect Testing System; C-S, Cross-sectional; EDT, Emotion Detection task; E60, Ekman60; EP, Emotional Processing; EPT, Empathy for Pain task; FBT, False belief task; FH, Frith-Happé animations task; FIT, Facial Identification task; FPT, Faux paux test; G, Good; G1, group 1; G2, group 2; GRN-PPA, progranulin-associated aphasia; HC, Healthy Controls; IGT, Iowa Gambling Task; IRI, Interpersonal Reactivity Index; JBI, Joanna Briggs Institute Critical Evaluation Checklists; Le, Left; L, Low; Lo, Longitudinal; lvPPA, logopenic variant PPA; M, Moderate; MET, Multifaceted Empathy Test; mini-SEA, mini Social cognition and Emotion Assessment; N/A, not available; ND, neurodegenerative dementia; nfvPPA, Non fluent variant PPA; RMET, Reading the Mind in the Eyes Test; R, Right; SET, test Storey-based Empathy Task; SNQ, Social Norms Questionnaire; SP, Social Perception; svPPA, semantic variant PPA; TASIT, The Awareness of Social Inference Test; ToM, Theory of Mind; TRCT, The Referential Communication Task*.

**Table 3:** Summary of studies on social cognition in the primary progressive aphasia.

**References**	**Design**	**Type of PPA**	**Patients (*N*)**	**Mean age of patients (S.D)**	**HC (*N*)**	**Mean age of HC (S.D)**	**SC domains**	**Assessment tools**	**Results**	**JBI**
Rohrer et al. (2012)	C-S	nfvPPA, lvPPA, GRN-PPA	19	68.6 (7.9)	14	68.2 (4.8)	EP	*Ad-hoc* test	All PPA worse than controls	G
Irish et al. (2013)	C-S	svPPA	Le: 12; R: 10	Le: 64.9 (7.0); R: 68.0 (6.7)	20	67.7 (5.3)	EP	TASIT, IRI, FIT, ET	Right svPPA worse than left svPPA	G
Binney et al. (2016)	C-S	svPPA	33	62 (7.2)	14	66.6 (4.2)	EP	CATS, TASIT, IRI	svPPA worse than controls	G
Bejanin et al. (2017)	C-S	svPPA	19	66.9 (6.97)	36	64.14 (8.25)	ToM	FBT	svPPA worse than controls	G
Zahn et al. (2017)	C-S	svPPA, bvFTD	19	67.3 (8.3)	19	60.7 (9.1)	SP	*Ad-hoc* test	svPPA and bvFTD worse than controls	G
Multani et al. (2017)	C-S	svPPA, nfvPPA, lvPPA	13, 11, 9	64.0 (7.22); 68.7 (6.79); 61.6 (6.69)	32	67.0 (4.39)	EP	TASIT, *Ad-hoc* test	All PPA worse than controls	G
Hazelton et al. (2017)	C-S	lvPPA	16	67.3 (7.6)	24	67.9 (6.8)	EP	IRI	No significant difference	M
Bertoux et al. (2020)	C-S	svPPA	16	67.89 (6.7)	20	63.26 (6.8)	ToM, EP	ToM-15, ADFES	svPPA worse than controls in EP	L

*AB, Attribution Bias; ADFES, Amsterdam Dynamic Facial Expression Set; CATS, Affect Matching subtest of the Comprehensive Affect Testing System; C-S, Cross-sectional; EDT, Emotion Detection task; E60, Ekman60; EP, Emotional Processing; EPT, Empathy for Pain task; FBT, False belief task; FH, Frith-Happé animations task; FIT, Facial Identification task; FPT, Faux paux test; G, Good; G1, group 1; G2, group 2; GRN-PPA, progranulin-associated aphasia; HC, Healthy Controls; IGT, Iowa Gambling Task; IRI, Interpersonal Reactivity Index; JBI, Joanna Briggs Institute Critical Evaluation Checklists; Le, Left; L, Low; Lo, Longitudinal; lvPPA, logopenic variant PPA; M, Moderate; MET, Multifaceted Empathy Test; mini-SEA, mini Social cognition and Emotion Assessment; N/A, not available; ND, neurodegenerative dementia; nfvPPA, Non fluent variant PPA; RMET, Reading the Mind in the Eyes Test; R, Right; SET, test Storey-based Empathy Task; SNQ, Social Norms Questionnaire; SP, Social Perception; svPPA, semantic variant PPA; TASIT, The Awareness of Social Inference Test; ToM, Theory of Mind; TRCT, The Referential Communication Task*.

**Table 4:** Summary of studies on social cognition in the Alzheimer's disease vs. behavioural variant frontotemporal dementia.

**References**	**Design**	**AD patients (*N*)**	**Mean age of AD (s.d)**	**bvFTD patients (*N*)**	**Mean age of bvFTD (S.D)**	**HC (N)**	**Mean age of HC (S.D)**	**SC domains**	**Assessment tools**	**Results**	**JBI**
Fernandez-Duque et al. (2009)	C-S	17	69.4 (5.7)	11	60.6 (7.2)	12	68.7 (8.8)	ToM	*Ad-hoc*	No significant results	L
Kipps et al. (2009b)	C-S	9	69.0 (6.9)	26	62.25 (7.15)	16	66.4 (4.9)	SP	TASIT	bvFTD worse than AD	G
Kipps et al. (2009a)	C-S	14	67.5 (9.0)	14	63.2 (8.0)	16	N/A	EP	EH	bvFTD worse than AD	M
Fernandez-Duque et al. (2010)	C-S	8	67.7 (6.6)	9	62.3 (6.7)	10	65.4 (8.5)	EP	*Ad-hoc*	No significant results	M
Cova et al. (2012)	C-S	10	78.0 (8.9)	12	66.5 (10.2)	10	66.0 (7.1)	AB	*Ad-hoc*	No significant results	G
Le Bouc et al. (2012)	C-S	12	61.9 (1.8)	11	58.7 (1.5)	20	59.8 (1.5)	ToM	FBT	bvFTD worse in second-order FBT	G
Shany-Ur et al. (2012)	C-S	32	62.3 (9.1)	39	61.6 (7.3)	77	68.2 (8.9)	ToM	TASIT, UCSF, CATS	bvFTD worse than AD	G
Buhl et al. (2013)	C-S	10	66 (N/A)	11	67 (N/A)	N/A	N/A	ToM, EP, SP	RMET, EH, TASIT	bvFTD worse than AD	G
Freedman et al. (2013)	C-S	21	71.6 (13.3)	14	60.7 (7.4)	31	65.0 (11.4)	ToM	FBT, *Ad-hoc* test	bvFTD worse in second-order FBT	M
Kéri (2014)	C-S	20	66.2 (7.9)	16	58.9 (7.3)	20	60.1 (7.4)	ToM	RMET	bvFTD worse than AD	M
Kumfor et al. (2014a)	Lo	17	67.4 (7.8)	20	66.6 (9.6)	24	67.9 (6.2)	EP, SP	E60, TASIT	All patients with decline	M
Bertoux et al. (2015)	C-S	33	71.6 (9.9)	60	66.1 (8.8)	30	66.2 (9.9)	EP	SEA	bvFTD worse than AD	M
Chiu et al. (2016)	C-S	21	70.21 (10.8)	25	66.0 (9.0)	31	68.4 (8.2)	EP	*Ad-hoc* test	bvFTD worse than AD	G
Dermody et al. (2016)	C-S	24	66.1 (8.0)	25	63.0 (8.7)	22	68.2 (6.7)	EP	IRI, E60	bvFTD worse than AD	G
Dodich et al. (2016)	C-S	12	73.17 (10.05)	20	66.8 (8.66)	65	66.89 (8.66)	ToM	SET	bvFTD worse than AD	G
Carr et al., 2017	C-S	12	59.25 (4.74)	12	62.29 (9.64)	N/A	N/A	EP	EQ	bvFTD worse than AD	G
Fong et al. (2017a)	C-S	11	61.36 (5.70)	10	62.40 (11.51)	9	53.88 (9.51)	EP	MBI, SNQ	bvFTD worse than AD	M
Fong et al. (2017b)	C-S	11	61.36 (5.70)	11	62.91 (11.05)	12	54.17 (9.80)	AB	SACS	bvFTD worse than AD	M
Kumfor et al. (2017)	C-S	23	66.1 (7.8)	25	65 (8.6)	25	64.8 (5.9)	SP	TASIT-S	bvFTD worse than AD	G
Ramanan et al. (2017)	C-S	29	71.5 (9.6)	44	65.25 (9.39)	44	65.25 (9.39)	ToM	Mini-SEA, FPT	bvFTD worse than AD	M
Reul et al. (2017)	C-S	43	72.0 (9.0)	26	65.0 (8.0)	26	65.0 (8.0)	EP	SEA	No significant results	G
Santamaria-Garcia et al. (2017)	C-S	24	63.1 (5.64)	20	5.9 (6.35)	20	61.1 (7.98)	ToM	RMET	bvFTD worse than AD	G
Sturm et al. (2017)	C-S	15	65.1 (10.2)	20	63.9 (6.7)	39	70.0 (5.0)	EP	IRI, *Ad-hoc*	bvFTD worse than AD	G
Wong et al. (2017)	C-S	14	68.06 (8.52)	20	62.23 (8.03)	20	63.29 (6.53)	SP	*Ad-hoc*	No significant results	M
Carr et al. (2018)	C-S	8	60.0 (4.9)	8	61.3 (10.1)	8	59.0 (5.1)	EP	*Ad-hoc*	bvFTD worse than AD	G
Dodich et al. (2018)	C-S	47	7.98 (9.92)	48	68.24 (8.57)	N/A	N/A	EP	E60, SNQ	bvFTD worse than AD	G
Synn et al. (2018)	C-S	18	68.9 (8.2)	18	63.1 (8.8)	25	66.8 (5.5)	ToM, EP	FH, IRI	No significant results	G
Sturm et al. (2018)	C-S	25	62.0 (5.9)	30	63.5 (8.4)	25	67.4 (5.9)	EP	*Ad-hoc*	bvFTD worse than AD	G

*AB, Attributional bias; AD, Alzheimer's Disease; bvFTD, behavioural variant of Frontotemporal Dementia; CATS, The comprehensive affect testing system; C-S, Cross-sectional; E60, Ekman 60; EH, Emotion Hexagon; EP, Emotional Processing; EQ, The Schutte Self Report Emotional Intelligence; FH, Frith-Happé animations task; FBT, False belief task; FPT, Faux pas test; G, Good; HC, Healthy Controls; IRI, The interpersonal reactivity index; JBI, Joanna Briggs Institute Critical Evaluation Checklists; L, Low; Lo, Longitudinal; M, Moderate; MBI, The Moral Behaviour Inventory; mini-SEA, mini Social cognition and Emotion Assessment; N/A, not available; RMET, Reading the Mind in the Eyes Test; SACS, The Social Attribution Coding System; SC, Social Cognition; SEA, Ekman Facial Emotion Test; SET, Storey-based Empathy Task; SNQ, the social norms questionnaire; SP, Social Perception; TASIT, The Awareness of Social inference Test; UCSF, cognitive Theory of Mind Test; ToM, Theory of Mind*.

**Table 5:** Summary of studies on social cognition in Alzheimer's disease vs. primary progressive aphasia.

**References**	**Design**	**AD patients (*N*)**	**Mean age of AD (S.D)**	**Type of PPA**	**PPA patients (*N*)**	**Mean age of PPA (S.D)**	**HC (N)**	**Mean age of HC (S.D)**	**SC domains**	**Assessment tools**	**Results**	**JBI**
Rankin et al. (2009)	C-S	27	59.2 (7.0)	svPPA	11	63.0 (8.6)	13	61.8 (10.3)	EP, SP	TASIT, CATS	svPPA worse than AD	G
Goodkind et al. (2012)	C-S	15	59.8 (5.3)	nfvPPA, svPPA	3,13	67.0 (9.8), 63.9 (8.2)	10	N/A	EP	*Ad-hoc*	No significant results	G
Miller et al. (2013)	C-S	35	66.1 (8.5)	nfvPPA, svPPA	8, 16	63.1 (8.8)	N/A	N/A	EP	FAST	No significant results	G
Narme et al. (2013)	C-S	13	74.5 (8.3)	svPPA	13	62.3 (6.9)	26	68.9 (9.6)	EP, ToM	E60, FPT, IRI	svPPA worse than AD	G
Hutchings et al. (2015)	C-S	10	66.25 (9.10)	svPPA	15	64.05 (5.85)	17	70.78 (4.80)	EP	SEQ	svPPA worse than AD	G
Kumfor et al. (2016)	Lo	33	65.1 (7.8)	svPPA	31	62.2 (6.8)	25	64.3 (4.0)	EP	SEA	svPPA with greater decline than AD	M
Park et al. (2017)	C-S	32	76.75 (8.47)	svPPA	13	71.92 (3.64)	33	70.97 (6.45)	EP	E60	svPPA worse than AD	G

*AD, Alzheimer's Disease; CATS, Comprehensive Affect Testing System; C-S, Cross-sectional; E60, Ekman 60 task; EC, Ekman Caricatures; EM, Emotional Processing; FADT, Face Affect Discrimination Task; FAST, Facial Affect Selection test; FERT, Facial emotion recognition; FIDT, Face Identify Discrimination Task; FPT, Faux Pas test; FTD, Frontotemporal Dementia; G, Good; HC, Healthy Controls; IRI, Interpersonal Reactivity Index; JBI, Joanna Briggs Institute Critical Evaluation Checklists; L, Low; Lo, Longitudinal; LBD, Lewy Body Dementia; M, Moderate; mini-SEA, Mini-Social Cognition & Emotional Assessment; N/A, not available; nfvPPA, non-fluent variant PPA; PPA, Primary Progressive Aphasia; RMET, Reading the mind in the eyes test; SC, Social Cognition; SEA, Facial Emotion Recognition Test; SEQ, Socio-Emotional Questionnaire; SET, The Storey-based Empathy Task; SP, Social Perception; svPPA, semantic variant PPA; TASIT, The Awareness of Social Inference Test; ToM, Theory of Mind*.

**Table 6:** Summary of studies on social cognition in behavioural variant frontotemporal dementia vs. primary progressive aphasia.

**References**	**Design**	**bvFTD patient (*N*)**	**Mean age of bvFTD (S.D)**	**Type of PPA**	**PPA patients (*N*)**	**Mean age of PPA (S.D)**	**HC (*N*)**	**Mean age of HC (S.D)**	**SC domains**	**Assessment tools**	**Results**	**JBI**
Eslinger et al. (2011)	C-S	12	N/A	nfvPPA, svPPA	7, 7	N/A	16	N/A	EP, ToM	IRI, *Ad-hoc* test	bvFTD worse than PPA	M
Kumfor et al. (2011)	C-S	16	61.5 (9.7)	nfvPPA, svPPA	13, 12	65.5 (11.4), 62.4 (8.8)	37	64.6 (4.5)	EP	E60, EC	No significant results	M
Omar et al. (2011)	C-S	16	64.7 (8.0)	svPPA	10	62.4 (8.8)	21	67.0 (8.8)	EP	*Ad-hoc* test	No significant results	M
Couto et al., 2013	C-S	12	69.8 (7.3)	nfvPPA	10	64.9 (8.6)	18	69.8 (7.3)	ToM, EP	RMET, FERT	No significant results	M
Hsieh et al. (2013)	C-S	18	63.7 (7.4)	svPPA	14	64.3 (8.5)	30	68.1 (5.6)	EP	IRI, *Ad-hoc* test	bvFTD worse than svPPA	M
Irish et al. (2014)	C-S	10	63.6 (7.3)	svPPA	11	63.4 (6.0)	14	68 (8.0)	ToM	*Ad-hoc* test	No significant results	L
Sollberger et al. (2014)	C-S	28	62.4 (8.2)	nfvPPA, svPPA	4, 16	62.0 (9.4), 61.8 (6.7)	19	71.3 (7.5)	EP	IRI	No significant results	G
Clark et al. (2015a)	C-S	22	67 (7.7)	svPPA	11	67 (7.7)	21	66 (5.0)	EP	*Ad-hoc* test	No significant results	M
Clark et al. (2015b)	C-S	15	65 (7.3)	nfvPPA, svPPA	10, 7	69.4 (7.4), 66.9 (6.2)	21	65.9 (5.0)	EP	*Ad-hoc* test	bvFTD and svPPA worse than nfvPPA	G
Downey et al. (2015)	C-S	29	64 (7.1)	svPPA	15	65 (6.6)	37	63 (7.8)	EP	TASIT	No significant results	G
Kamminga et al. (2015)	C-S	19	60.5 (8.5)	svPPA	12	65 (7.6)	20	65.8 (6.2)	EP	*Ad-hoc* test, E60	No significant results	G
Chen et al. (2018)	C-S	45	60.9 (8.0)	svPPA, nfvPPA	28, 23	62.0 (6.2), 65.4(10.7)	35	64.4 (5.6)	EP	FIDT, FADT, FAST	bvFTD worse than nfvPPA	G
Kumfor et al. (2018a)	C-S	25	60.7 (6.6)	svPPA	14	64.7 (7.1)	24	65.2 (6.8)	EP	*Ad-hoc* test	No significant results	L
Kumfor et al. (2018b)	C-S	19	62.7 (8.7)	svPPA	12	64.9 (8.3)	20	66.3 (6.1)	EP	*Ad-hoc* test	No significant results	M
Marshall et al. (2018)	C-S	19	66.2 (6.3)	svPPA, nfvPPA	9, 9	66.1 (6.5), 69.6 (6.5)	21	69.1 (5.3)	EP	*Ad-hoc* test	No significant results	M

*AD, Alzheimer's Disease; CATS, Comprehensive Affect Testing System; C-S, Cross-sectional; E60, Ekman 60 task; EC, Ekman Caricatures; EM, Emotional Processing; FADT, Face Affect Discrimination Task; FAST, Facial Affect Selection test; FERT, Facial emotion recognition; FIDT, Face Identify Discrimination Task; FPT, Faux Pas test; FTD, Frontotemporal Dementia; G, Good; HC, Healthy Controls; IRI, Interpersonal Reactivity Index; JBI, Joanna Briggs Institute Critical Evaluation Checklists; L, Low; Lo, Longitudinal; LBD, Lewy Body Dementia; M, Moderate; mini-SEA, Mini-Social Cognition & Emotional Assessment; N/A, not available; nfvPPA, non-fluent variant PPA; PPA, Primary Progressive Aphasia; RMET, Reading the mind in the eyes test; SC, Social Cognition; SEA, Facial Emotion Recognition Test; SEQ, Socio-Emotional Questionnaire; SET, The Storey-based Empathy Task; SP, Social Perception; svPPA, semantic variant PPA; TASIT, The Awareness of Social Inference Test; ToM, Theory of Mind*.

**Table 7:** Summary of studies on social cognition in unspecific frontotemporal dementia.

**References**	**Design**	**FTD patients (*N*)**	**Mean age of FTD (S.D)**	**HC (N)**	**Mean age of HC (S.D)**	**SC domains**	**Assessment tools**	**Results**	**JBI**
Bedoin et al. (2009)	C-S	11	61.2 (3.2)	11	58.54 (5.3)	EP	*Ad hoc* test	Patients with impairment in EP	M
Zahn et al. (2009)	C-S	29	61.3 (8.9)	15	61.5 (8.5)	SP	*Ad hoc* test	Patients with impairment in SP	L
Bediou et al. (2009)	C-S	10	67.0 (7.0)	10	70.0 (6.0)	EP	*Ad hoc* test	FTD worse than AD	M
Formica et al. (2020)	C-S	14	74.29 (4.68)	N/A	N/A	ToM	RMET; SET	FTD worse than AD	M
Russell et al. (2020)	C-S	103	N/A	246	46 (12.8)	ToM; EP	FPT; FERT	FTD worse than controls	G

*AD, Alzheimer Disease; CATS, Comprehensive Affect Testing System; C-S, Cross-sectional; E60, Ekman 60 task; EC, Ekman Caricatures; EM, Emotional Processing; FADT, Face Affect Discrimination Task; FAST, Facial Affect Selection test; FERT, Facial emotion recognition; FIDT, Face Identify Discrimination Task; FPT, Faux Pas test; FTD, Frontotemporal Dementia; G, Good; HC, Healthy Controls; IRI, Interpersonal Reactivity Index; JBI, Joanna Briggs Institute Critical Evaluation Checklists; L, Low; Lo, Longitudinal; LBD, Lewy Body Dementia; M, Moderate; mini-SEA, Mini-Social Cognition & Emotional Assessment; N/A, not available; nfvPPA, non-fluent variant PPA; PPA, Primary Progressive Aphasia; RMET, Reading the mind in the eyes test; SC, Social Cognition; SEA, Facial Emotion Recognition Test; SEQ, Socio-Emotional Questionnaire; SET, The Story-based Empathy Task; SP, Social Perception; svPPA, semantic variant PPA; TASIT, The Awareness of Social Inference Test; ToM, Theory of Mind*.

**Table 8:** Summary of studies on social cognition in lewy body dementia.

**References**	**Design**	**LBD patients (*N*)**	**Mean age of LBD (S.D)**	**HC (*N*)**	**Mean age of HC (S.D)**	**SC domains**	**Assessment tools**	**Results**	**JBI**
Heitz et al. (2016)	C-S	33	68.0 (8.4)	16	68.3 (10.5)	EP, ToM	E60, FPT, RMET	Patients impaired in ToM	G
Kemp et al. (2017)	C-S	37	67.19 (8.64)	29	68.79 (7.9)	EP, ToM	mini-SEA, RMET	Patients impaired in ToM	G

*AD, Alzheimer's Disease; CATS, Comprehensive Affect Testing System; C-S, Cross-sectional; E60, Ekman 60 task; EC, Ekman Caricatures; EM, Emotional Processing; FADT, Face Affect Discrimination Task; FAST, Facial Affect Selection test; FERT, Facial emotion recognition; FIDT, Face Identify Discrimination Task; FPT, Faux Pas test; FTD, Frontotemporal Dementia; G, Good; HC, Healthy Controls; IRI, Interpersonal Reactivity Index; JBI, Joanna Briggs Institute Critical Evaluation Checklists; L, Low; Lo, Longitudinal; LBD, Lewy Body Dementia; M, Moderate; mini-SEA, Mini-Social Cognition & Emotional Assessment; N/A, not available; nfvPPA, non-fluent variant PPA; PPA, Primary Progressive Aphasia; RMET, Reading the mind in the eyes test; SC, Social Cognition; SEA, Facial Emotion Recognition Test; SEQ, Socio-Emotional Questionnaire; SET, The Storey-based Empathy Task; SP, Social Perception; svPPA, semantic variant PPA; TASIT, The Awareness of Social Inference Test; ToM, Theory of Mind*.

### Social Cognition in AD

Thirty-five studies with samples from patients with AD were selected. All but two longitudinal studies (Torres et al., 2015; Antonio Garcia-Casal et al., 2017) used a cross-sectional design. Based on the different domains of social cognition, we observed that 18 studies reported performance on ToM tasks: five studies (28%) observed a similar performance for patients with AD and healthy controls on first-order cognitive ToM tasks (those in which subjects must recognise the beliefs of others) and affective ToM tasks (Henry et al., 2009; Castelli et al., 2011; Choong and Doody, 2013; Laisney et al., 2013; El Haj et al., 2015). However, ten studies (56%) indicated that patients with AD showed impairments on complex tasks when compared to healthy controls, specifically on second-order tasks (those in which subjects must recognise false beliefs or intentions of others) (Youmans and Bourgeois, 2010; Maki et al., 2013a; Fliss et al., 2016; Moreau et al., 2016; Duclos et al., 2018b; Perri et al., 2018; Takenoshita et al., 2018; El Haj et al., 2019; Lozachmeur et al., 2019; Chainay and Gaubert, 2020). Furthermore, three studies (16%) reported that a deterioration of ToM progresses with the disease (Yamaguchi et al., 2012, 2019; Maki et al., 2013b).

Thirteen articles addressed emotional processing, ten of which (77%) indicated that the patients showed a deterioration in this domain compared to the healthy controls (Kumfor et al., 2014a; Insch et al., 2015; Torres et al., 2015; Sava et al., 2017, 2019; Daley et al., 2018; Antonio Garcia-Casal et al., 2019; Arroyo-Anlló et al., 2019; Dourado et al., 2019; Hayashi and Terada, 2021). However, two studies showed a similar performance in both samples (patients and healthy controls) (Garcia-Rodriguez et al., 2009; Guaita et al., 2009). We highlight here the longitudinal study by Antonio Garcia-Casal et al. (2017), which observed an improvement in this domain after a training program (Antonio Garcia-Casal et al., 2017).

Three studies (100%) on social perception found that this domain deteriorated in patients with AD compared to healthy controls (Insch et al., 2017; Poveda et al., 2017; Simm et al., 2017). A similar finding was observed for attribution bias; Moyse et al. (2015) found that patients showed an impairment when inferring the age of other people (Moyse et al., 2015) (Table 1).

### Social Cognition in bvFTD

Twenty-three selected articles studied groups of patients with bvFTD. Fifteen of these works studied the ToM domain: 13 employed a transverse design, and two used a longitudinal design. Among the first group, 11 (85%) studies documented significantly lower scores for patients than for healthy controls (Torralva et al., 2009, 2015; Grossman et al., 2010; Custodio et al., 2015; Sedeno et al., 2016; Tabernero and Politis, 2016; Tabernero et al., 2017; Schroeter et al., 2018; Giovagnoli et al., 2019; Van den Stock et al., 2019; Lillo et al., 2020). The findings of the longitudinal studies were contradictory, since one study found that patients with bvFTD showed a greater deterioration than patients with other types of dementia (Gossink et al., 2018), while the other study did not observe changes in ToM over time (Reus et al., 2018).

Fourteen articles aimed to study emotional processing: ten (71%) reported impairments in this domain in patients compared to healthy subjects (Baez et al., 2014; Savage et al., 2014; Cerami et al., 2015; Oliver et al., 2015; Van den Stock et al., 2015, 2017; Jastorff et al., 2016; Tabernero et al., 2017; Kawano et al., 2020; Lillo et al., 2020). Additionally, patients with bvFTD were more affected than patients with other unspecified dementias (Gossink et al., 2018; Reus et al., 2018).

Three studies addressed the attribution bias domain, all of which showed worse performance for patients with bvFTD than healthy controls (Cerami et al., 2014; Baez et al., 2016b; Van den Stock et al., 2017) (Table 2).

### Social Cognition in PPA

Eight articles studied social cognition in patients with PPA, all with a cross-sectional design and a control group. Four of these studies included patients with svPPA, one of which documented deterioration in ToM compared to healthy controls (Bejanin et al., 2017) and three studies (75%) reported impairments in emotional processing (Irish et al., 2013; Binney et al., 2016; Bertoux et al., 2020). Two studies were conducted with samples of patients with nfvPPA, and both observed deficits in emotional processing; one reported impairments in an emotional prosody task (Rohrer et al., 2012), and the other reported impairments in an empathy test. Additionally, these authors observed that scores on the social cognition test were significantly worse after the onset of the disease than before (Hazelton et al., 2017). Multani et al. evaluated emotional processing in a sample that included patients with every type of PPA. All three groups performed significantly worse than controls, with patients with svPPA showing the lowest accuracy in emotion recognition (Multani et al., 2017). The study by Zahn et al. included a sample of patients with svPPA, bvFTD and mixed diagnoses. Patients scored significantly worse than healthy controls on social perception tasks (Zahn et al., 2017) (Table 3).

### Social Cognition in AD Compared With bvFTD

Twenty-eight studies compared the social cognition of patients with AD with patients with bvFTD. Ten of these studies evaluated ToM, eight of which (80%) found a specific impairment in patients with bvFTD that was independent of general cognition (Le Bouc et al., 2012; Shany-Ur et al., 2012; Buhl et al., 2013; Freedman et al., 2013; Kéri, 2014; Dodich et al., 2016; Ramanan et al., 2017; Santamaria-Garcia et al., 2017). The remaining two studies did not report significant differences in ToM between patients with AD and bvFTD (Fernandez-Duque et al., 2009; Synn et al., 2018).

Fourteen studies explored emotional processing and empathy. Eleven studies (79%) reported a greater deficit in individuals with bvFTD compared with patients with AD (Kipps et al., 2009a; Buhl et al., 2013; Bertoux et al., 2015; Chiu et al., 2016; Dermody et al., 2016; Carr et al., 2017, 2018; Fong et al., 2017a; Sturm et al., 2017, 2018; Dodich et al., 2018), and the other three studies specifically observed reduced levels of empathy indicators in patients with bvFTD. The remaining studies did not report significant differences between the two groups of patients (Fernandez-Duque et al., 2010; Reul et al., 2017; Synn et al., 2018).

Of four studies that included social perception in their analyses, three (75%) found that patients with bvFTD had greater deficits than patients with AD (Kipps et al., 2009b; Buhl et al., 2013; Kumfor et al., 2017), but one did not detect significant differences (Wong et al., 2017).

Two articles explored attribution bias; one observed similar performance in patients with both types of dementia (Cova et al., 2012), while the other reported a specific animacy attribution impairment in patients with bvFTD (Fong et al., 2017b).

Finally, a longitudinal study reported that both patients with AD and bvFTD exhibited decreased emotional processing and social perception in the long term (Kumfor et al., 2014b) (Table 4).

### Social Cognition in AD Compared With PPA

Seven studies compared social cognition between patients with AD and patients with PPA (svPPA and nfvPPA). Emotional processing and empathy were studied in six cross-sectional studies. In four of these studies (67%), poorer performance was observed for patients with svPPA than patients with AD (Rankin et al., 2009; Narme et al., 2013; Hutchings et al., 2015; Park et al., 2017). The study by Park et al. showed deficits in the recognition of emotion of negative valence; however, this phenomenon was not replicated for positive emotions. In contrast, the remaining two studies found no difference between the groups compared (Goodkind et al., 2012; Miller et al., 2013). Furthermore, a longitudinal study conducted over 6 years reported that patients' performance in emotional processing changed over time. A significant interaction between time and diagnosis was observed in an emotional processing task where both patients with left and right svPPA decreased at a faster rate than patients with AD (Kumfor et al., 2016).

Social perception was studied in the study by Rankin and colleagues. They compared patients with AD and svPPA, concluding that the svPPA group was affected in the ability to interpret naturalistic social interactions, such as sarcasm (Rankin et al., 2009) (Table 5).

### Social Cognition in bvFTD Compared With PPA

In this review, fifteen cross-sectional studies compared the social cognition of patients with two different variants of FTD, bvFTD and PPA, the second including the non-fluent (nfvPPA) and semantic (svPPA) subtypes. Only two studies examined ToM, and they found a similar performance of patients with both variants of FTD (Couto et al., 2013; Irish et al., 2014).

Fourteen papers focused on the study of emotional processing. Ten of these studies (71%) did not describe differences in emotional processing impairment between patients stratified according to the FTD subtype (Kumfor et al., 2011, 2018a,b; Omar et al., 2011; Couto et al., 2013; Sollberger et al., 2014; Clark et al., 2015a; Downey et al., 2015; Kamminga et al., 2015; Marshall et al., 2018). The last study focused especially on empathy (Sollberger et al., 2014). However, the other four articles reported intergroup differences. Chen et al. (2018) observed that patients with bvFTD performed worse on emotional processing tasks than patients with nfvPPA. Likewise, Clark et al. (2015b) reported that an altered sense of humour was particularly prominent in patients with bvFTD and svPPA compared to patients with nfvPPA. Two papers reported that patients with bvFTD were less empathetic than patients with svPPA (Eslinger et al., 2011; Hsieh et al., 2013).

All studies showed that patients with bvFTD, svPPA, and nfvPPA performed worse than controls on emotion recognition tasks (Table 6).

### Social Cognition in Unspecific Frontotemporal Dementia (UFD)

Five cross-sectional studies were unable to be included in the previous classifications. Two studies compared samples of patients with AD with patients with nonspecific FTD: Bediou et al. concluded that patients with AD exhibited better performance on an expression recognition task than a sample of patients with FTD (Bediou et al., 2009), while Formica et al. found that patients with AD had better performance than patients with FTD on a ToM subtest (Formica et al., 2020). Bedoin et al. (2009) did not specify what type of FTD was present in the group of patients examined. Their results showed that patients performed worse than controls on two implicit emotional processing tasks. The study by Zahn et al. (2009) was performed in a sample of patients with bvFTD, nfvPPA and svPPA. Patients showed significantly worse performance than controls on a social perception task. The last of the cross-sectional studies included a sample of patients with bvFTD, PPA and other FTDs, all of whom were carriers of some genetic mutation related to familial FTD. Patients showed worse performance than controls on ToM and emotional processing tasks, regardless of clinical diagnosis. Furthermore, they also showed worse performance on the same tasks than a presymptomatic group of genetic mutation carriers (Russell et al., 2020) (Table 7).

### Social Cognition in DLB

Two cross-sectional studies were performed on samples of patients with DLB. Both compared their samples of patients to healthy controls. In one study, patients showed significantly worse performance than controls on a ToM task (Kemp et al., 2017). In the other study, a group of patients with AD was also included in the analyses, but only patients with DLB showed poorer performance on the ToM task than controls. With respect to emotional processing, no differences were observed between the three groups (Heitz et al., 2016) (Table 8).

### Quality Assessment

Using the JBI tools to identify the strengths and weaknesses of a research article, each study included in this review was individually assessed. Predetermined cutoff scores were established prior to the start of the assessment process. The quality of the articles was considered good when more than 75% of the criteria were met, moderate when it met between 50–75%, and low when it met less than 50%. The complete process was performed by four reviewers (ESS, NMG, GAF, and MSR). Subsequently, a random sample of 20% of the studies was re-evaluated. The reviewers discussed any discrepancies in the evaluation process to reach a consensus. Of the 109 studies with a case-control design, 7 were low quality, 48 were moderate quality, and 54 studies were high quality. Of the 6 studies with a longitudinal design, 5 were moderate quality and one was high quality. Of the 8 cross-sectional studies, 5 were of moderate quality, and 3 were of high quality. Taking all studies together, 6% were low quality (*N* = 7), 47% were moderate quality (*N* = 58) and 47% were high quality (*N* = 58) (Supplementary Tables 1–3).

## Discussion

We conducted this systematic review to examine the components of social cognition across different aetiologies of dementia. One hundred twenty-two studies that met the inclusion criteria were summarised. These data showed different patterns of impairment in ToM, emotional processing, social perception, and attribution bias across patients with dementia caused by AD, FTD, and DLB, which are discussed below.

### Social Cognition in AD

The weight of the studies on patients with AD in this review is worth noting, most of which agree that an impairment in social cognition is not as evident in these patients when compared to healthy controls (Yamaguchi et al., 2012, 2018; Maki et al., 2013b). Cognitive impairment in individuals with AD is characterised by an insidious onset and gradual progression, with memory and executive functions representing the first affected processes (Elamin et al., 2012; Hugo and Ganguli, 2014). This neuropsychological profile is potentially associated with the relative preservation of social cognition in the early stages of the disease. This finding may explain the normal performance of patients with AD observed on some social cognition tasks, suggesting that the deterioration of this domain would be secondary to the decline of other cognitive functions.

In the ToM domain, when patients with AD were compared to healthy controls, both exhibited similar performance on cognitive ToM simple order tasks (Choong and Doody, 2013; Laisney et al., 2013). However, when the level of complexity increased (in second-order tasks), patients with AD presented a deterioration in their ToM performance (Castelli et al., 2011; Maki et al., 2013a; El Haj et al., 2015; Fliss et al., 2016; Moreau et al., 2016; Duclos et al., 2018b; Perri et al., 2018; Takenoshita et al., 2018). This finding is consistent with the results reported in previous systematic reviews explaining that advanced-level ToM skills are the first to be affected in individuals with AD (Elamin et al., 2012; Sandoz et al., 2014). These complex abilities require the operation of other processes, such as executive functions, which, when impaired, could contribute to poor performance on ToM tasks. Therefore, we cannot rule out that the impairment in these more complex ToM tasks is a direct consequence of a deficit on executive functions and not a pure impairment on social cognition. In this vein, Sandoz's review (Sandoz et al., 2014) indicates that some studies observed correlations between inhibition and ToM performance (Bailey and Henry, 2008; Li et al., 2013) and others found a strong relationship between flexibility and ToM ability (Phillips et al., 2011; Li et al., 2013). However, it should also be noted that not all studies reached these results (Bernstein et al., 2011; Cavallini et al., 2013) and therefore would not support the idea that the deterioration in ToM is due to deficiencies in executive functions.

Regarding emotional processing, no agreement was achieved among the different studies. However, the lack of consistency between studies based on different types of emotions should be noted. On the one hand, some studies reported that performance on emotional processing tasks was similar between AD groups and healthy control groups. Specifically, this similar performance was observed for face recognition tasks, in which the subjects were required to detect the emotions of happiness and surprise in the faces of others (Garcia-Rodriguez et al., 2009; Guaita et al., 2009). On the other hand, other authors identified differences in the recognition of negative emotions such as anger and sadness, with significantly lower scores for patients with AD (Lee et al., 2013; Kumfor et al., 2014a; Insch et al., 2015; Sava et al., 2017, 2019; Daley et al., 2018; Antonio Garcia-Casal et al., 2019). Several reviews on this topic have been published. In the meta-analysis of Klein-Koerkamp et al., the ability to decode emotions from faces was relatively preserved in patients with AD compared to healthy subjects (Klein-Koerkamp et al., 2012). McLellan et al. reported in his review that patients with AD exhibit poor recognition of sad facial expressions (McLellan et al., 2008), consistent with our review. Importantly, one study included in the present review concluded that patients with AD can improve their performance in this domain with a training program (Antonio Garcia-Casal et al., 2017). According to the authors, this is the first study reporting a rehabilitative treatment of facial emotion recognition in people with AD. However, they did not carry out a follow-up assessment to determine the duration of the effect of the therapy, which limits their results. Without question, the results are encouraging and open a path of work focused on prolonging the quality of life of AD patients over time through the improvement of the quality of interpersonal relationships.

Similar to the ToM domain, the social perception domain showed differences between patients with AD and healthy controls when performing complex tasks. Namely, people with AD performed comparably to a control group when following gaze. However, when the complexity increased and the task consisted of making explicit discrimination distinctions between direct and averted gaze, patients with AD recorded worse scores than controls (Insch et al., 2017; Poveda et al., 2017). Individuals with AD are at increased risk of misunderstanding their social world, including communicating and interacting with others, potentially due to their reduced ability to understand their own mental world and to interpret and reflect on thoughts, feelings, and beliefs about themselves, as well as their reduced ability to reflect on social relationships (Simm et al., 2017).

Abnormalities in the attributional bias domain were also observed, specifically when patients were required to infer other people's ages. Specifically, the profile of impairment depended on the age of the faces and the stage of the disease. Subjects with mild AD showed difficulties primarily in assessing the age of middle-aged adults. Subjects with moderate AD also presented difficulties estimating the age of young adult faces. However, both groups with mild and moderate AD were relatively good at estimating the age of older adults' faces, and they did not significantly differ from healthy controls (Moyse et al., 2015).

### Social Cognition in FTD

The present review confirmed that patients with different variants of FTD presented deficits in social cognition tasks compared to healthy controls. This finding is consistent with previous evidence indicating that social cognition is an ability that is particularly affected in individuals with FTD, along with language and executive function (Harciarek and Cosentino, 2013). Based on the current evidence, we were unable to clearly determine whether social cognition impairment is stable or deteriorates in the long term, since the vast majority of the studies analysed here employed a cross-sectional design. A longitudinal study included in the present review reported that patients with bvFTD show a decline in social cognition over time (Kumfor et al., 2014b). However, a deterioration of social cognition is not homogeneous between patients with different FTD subtypes, and thus, its longitudinal course may also vary.

Social cognition deterioration was found to be a core aspect of bvFTD in this review, since several studies comparing patients with different types of dementia reported that those with bvFTD present a more pronounced impairment in ToM and emotional processing. These findings correspond with a previous meta-analysis reporting a significant impairment in emotion recognition (Bora et al., 2016) in patients with bvFTD compared to healthy controls and patients with other types of dementia. Furthermore, the social cognition impairment in patients with bvFTD is a primary deficit and therefore does not underlie other neurocognitive deficits (Dodich et al., 2016). The social cognition deficits in patients with bvFTD might be caused by the specific degeneration of fronto-limbic networks, resulting in a particular emotional processing impairment that is not observable in patients with other types of dementia (Seelaar et al., 2011; Bora et al., 2016; Dodich et al., 2016). Neuroimaging studies could support this hypothesis, since specific relationships between certain brain areas belonging to these networks and different sub-processes of emotional processing have been suggested. For example, the feeling of empathy implies a process of affective sharing that is predominantly based on limbic structures such as the amygdala and the hippocampus (Carr et al., 2003; Decety and Chaminade, 2003; Vollm et al., 2006). Likewise, recognising that the source of emotion is outside oneself requires awareness and understanding of the emotion, which is based on frontal regions (Gallagher and Frith, 2003; Craig, 2009), specifically, recognising the separation between the self and the others is mediated by fronto-parietal circuits that involve the orbitofrontal cortex and the medial and dorsolateral prefrontal cortex (Decety and Sommerville, 2003; Saxe and Wexler, 2005; Decety and Lamm, 2007).

Although fewer studies examined social cognition in patients with PPA, they allowed us to obtain relevant findings. When comparing patients with svPPA, patients with nfvPPA and healthy controls, a social cognition deficit was confirmed in patients with both subtypes of FTD. Specifically, the social cognition impairment in individuals with svPPA (especially right svPPA) tends to be greater than that in those with nfvPPA and less than that in those with bvFTD. According to Fittipaldi et al. (2019), similarities between the svPPA and bvFTD profiles are explained by the fronto-temporal disruptions present in patients with both conditions. In summary, our findings are consistent with previous conclusions, proving that social cognition deficits are not exclusive to individuals with bvFTD, but they are frequent in patients with the language variant of FTD, including any of its subtypes, and can be observed in early stages of cognitive decline, particularly in patients with svPPA. In this case, single word comprehension problems or difficulty in naming objects in the early stages of svPPA might cause poor performance on social cognition tasks. However, the results of some studies do not support this hypothesis (Irish et al., 2013; Binney et al., 2016). In one of these studies, the authors found through covariate analysis that general processing impairments and semantic naming deficits did not explain marked emotion recognition deficits (Irish et al., 2013). Meanwhile, the other study found that patients with semantic dementia with predominantly left temporal atrophy showed deeper anomie, while patients with semantic dementia with predominantly right temporal atrophy showed greater difficulties in emotional processing. These results could indicate lack of relationship between semantic understanding and emotional processing (Binney et al., 2016).

The social cognition deficit in patients with FTD may affect their social interactions. In fact, according to Harciarek and Cosentino (2013), patients with bvFTD exhibit abnormal behaviours, such as eccentric, inappropriate or offensive behaviours, all of which compromise social interactions. Regarding the language variant of FTD, patients with PPA do not show abnormal behaviours at the onset of the disorder, although disrupted behaviours arise over time, such as aggressiveness or personal neglect (Harciarek and Cosentino, 2013).

### Comparison of Social Cognition Among Patients With Different Types of Neurodegenerative Diseases

We observed that all four domains of social cognition were more substantially affected in patients with FTD than in patients with AD. Emotional processing, social perception, and attribution bias were more impaired in patients with all FTD subtypes (bvFTD and PPA) than in patients with AD. Regarding ToM, most studies that compared individuals with different types of dementia found that patients with AD were the least affected. Specifically, in several studies, patients with bvFTD performed significantly worse on ToM tasks than patients with AD. These data coincide with a previous meta-analysis in which ToM was specifically studied in patients with bvFTD, patients with AD and healthy controls (Henry et al., 2014). Furthermore, the only study comparing patients with AD and patients with DLB also showed that the AD group was less affected, reporting no differences compared to controls, while the DLB group exhibited worse performance than healthy controls (Heitz et al., 2016).

Among social cognition domains, ToM has been the most widely studied. Several studies have indicated that patients with early-stage AD do not differ from healthy controls in this domain. However, as the disease progresses, ToM test performance worsens. An explanation for this finding is based on the accumulating evidence that the decrease in ToM performance is related to the age of the subjects (Maylor et al., 2002; Uekermann et al., 2006; McKinnon and Moscovitch, 2007; Slessor et al., 2007; Charlton et al., 2009; Duval et al., 2011), and due in part to a decrease in executive function, either caused by deficits in inhibitory control (German and Hehman, 2006; Bailey and Henry, 2008; Charlton et al., 2009) or difficulties in updating information in working memory (McKinnon and Moscovitch, 2007; Phillips et al., 2011).

Unlike patients with AD, in which ToM deficits appear to be related to the stage of dementia, patients with bvFTD show markedly decreased performance, particularly in the affective component, even in early stages of the disease (Torralva et al., 2015) when daily functioning is not affected and no other cognitive deficits are present. Therefore, these data support the concept proposed by several authors that the deterioration in ToM in patients with bvFTD is a central feature of this type of dementia, suggesting its evaluation as a differential diagnosis for bvFTD compared to other types of dementia. Notably, the type of test applied to measure ToM may play a determining role in establishing differences between individuals with FTD and AD. For instance, the performance on the Reading the Mind in the Eyes Test is similar in patients with both disorders, while in the Faux Pas test and sarcasm tasks, patients with FTD show poor performance compared to those with AD (Bora et al., 2016).

Overall, the different patterns of deterioration in social cognition described here might be explained by the neuropsychological profile of each neurodegenerative disease. In individuals with AD, the memory impairment beginning in the early stages might affect performance in social cognition and progressively worsen. In patients with FTD, the prominent compromise of executive functions might be associated with impairments in all components of social cognition, causing unadaptive social behaviour from the onset of the disease. Furthermore, these findings showed that social cognition is a neurocognitive domain whose functioning is altered by neurodegenerative processes, although differences are detected depending on the type of brain damage that differentially affects the components of social cognition.

### Tasks That Assess Social Cognition

Most of the studies included in this review used adapted and validated assessment tools to measure different social cognition domains. Among these instruments, the most frequently used was the Reading the Mind in the Eyes Test (RMET, used by 19 studies), followed by the False Belief Task (FBT, 15 studies), The Awareness of Social Inference Test (TASIT, 14 studies) and the Ekman 60 Faces Test (14 studies). However, it should be noted that ~33% of the studies (41 studies) included self-designed tasks (referred to as “*ad-hoc*” in Tables 1–8), which could affect the generalizability of their findings.

The RMET examines affective ToM (Baron-Cohen et al., 2001), since the subject must attribute another person's mental state. The RMET seems to be less demanding than other ToM tests (Lucena et al., 2020) and has been associated with the executive functions and the educational level (Heitz et al., 2016). Therefore, the RMET could be less sensitive to detect mild ToM deficits, which could explain why some studies have been unable to find differences between AD patients and controls. Moreover, the FBT assess first and second order false beliefs because individuals must inhibit their own knowledge of reality in order to recognise another person's false belief. The FBT may be sensitive to detect ToM alterations in dementia population, but since is related to memory performance, it is essential to control the possible confounding role of memory deficits (Lucena et al., 2020). Additionally, the TASIT assesses social perception by asking questions about of other people's intentions presented in short videos. This test has shown to be sensitive enough to identify alterations in the interpretation of emotions such as sarcasm in people with dementia (Kumfor et al., 2017). However, it requires an administration time between 60 and 90 min that could interfere with the performance of the subject. Finally, the Ekman 60 Test assesses the emotional recognition of facial expressions, and is sensitive to deficits in dementia (Strijkert et al., 2021). Furthermore, this test showed accuracy in distinguishing between AD and bvFTD patients (Dodich et al., 2021). However, the performance on this test may also be influenced by deficits in other cognitive domains, so these must be taken into account when interpreting its results.

In general, these commonly used tests have proven to be useful for measuring social cognition in population with different neurodegenerative diseases. However, the characteristics and limitations of each must be taken into account. First, it is important to be cautious when comparing the results provided by different tests, since they could measure different social cognition processes. These precautions are especially relevant when measuring the efficacy of interventions and clinical trials that must control the effect of possible covariates such as semantic processing, executive functions, memory or the presence of compensatory mechanisms as social reserve (Fliss et al., 2016). Regarding the social cognition domains, we found that tools for assessing attributional biases are less developed compared to other domains such as ToM or emotional processing. Most of the included studies that explored attributional biases did so through a task of inferring the age of other people, which is insufficient to know the patterns of individual attribution to social events.

### Strengths and Limitations

This review is subject to some limitations that are worth noting. Importantly, several limitations are inherent to each original study, as observed for the seven studies that exhibited low methodological quality. Furthermore, the existence of uncontrolled variables, such as progression of the disorder at the time of testing, the medications taken or comorbidities with other diseases, could not always be identified in the included articles; these variables may explain some of the apparent discrepancies across different studies. Another limitation of this review is publication bias, i.e., it did not take into account the grey literature, which may exclude studies with negative results. Future research must perform follow-up studies with larger samples. These investigations might provide more accurate information on social cognition in patients with different stages of the disorder. Another important and highly recommended aspect for future studies is the use of quality assessment tools that ensure reliability and validity, trying to reduce the heterogeneity of the tests used in the measurement of social cognition which limits the comparison and pooling of results.

Among the strengths of this work, this review represents the first effort to systematically summarise the state of social cognition (in all its domains) in patients with neurodegenerative diseases (in all types in which dementia is the main symptom) to the best of our knowledge.

## Conclusions

All domains of social cognition are affected in individuals with the different types of dementia studied. However, the degree of impairment differs, depending on the dementia type, the domain being investigated, and the evolution of the disease. The different performance patterns in social cognition observed in individuals with neurodegenerative diseases reveal that their functioning is sustained by a complex neurobiological system that, when compromised, affects the cognitive domains, preventing the individual from carrying out adaptive social behaviours.

## Data Availability Statement

The original contributions presented in the study are included in the article/Supplementary Material, further inquiries can be directed to the corresponding author/s.

## Author Contributions

ESS and RAA contributed to conception and design of the study. ESS organised the database. ESS, NMG, MSR, and GAF wrote sections of the manuscript and wrote the first draft of the manuscript. RAA and AP contributed to manuscript revision. All authors read and approved the submitted version.

## Funding

This work was supported by a Juan de la Cierva-Formación contract (ESS) from the Spanish Ministry of Science and Innovation (FJC2019-042390-I/AEI/10.13039/501100011033) and a Miguel Servet contract (RAA) from the Carlos III Health Institute (CP18/00003).

## Conflict of Interest

The authors declare that the research was conducted in the absence of any commercial or financial relationships that could be construed as a potential conflict of interest.

## Publisher's Note

All claims expressed in this article are solely those of the authors and do not necessarily represent those of their affiliated organizations, or those of the publisher, the editors and the reviewers. Any product that may be evaluated in this article, or claim that may be made by its manufacturer, is not guaranteed or endorsed by the publisher.
