# Corrigendum: Exploring the relationship between deficits in social cognition and neurodegenerative dementia: A systematic review

**DOI:** 10.3389/fnagi.2022.1031955

**Published:** 2022-09-26

**Authors:** Esther Setién-Suero, Nancy Murillo-García, Manuel Sevilla-Ramos, Georgelina Abreu-Fernández, Ana Pozueta, Rosa Ayesa-Arriola

**Affiliations:** ^1^Department of Psychiatry, School of Medicine, University of Cantabria, University Hospital Marqués de Valdecilla, Santander, Spain; ^2^IDIVAL, Valdecilla Biomedical Research Institute, Santander, Spain; ^3^Department of Psychology, Faculty of Health Sciences, University of Deusto, Bilbao, Spain; ^4^Neurology Service and Centro de Investigación Biomédica en Red sobre Enfermedades Neurodegenerativas (CIBERNED), Madrid, Spain; ^5^CIBERSAM, Biomedical Research Network on Mental Health Area, Madrid, Spain

**Keywords:** neurodegenerative disease, dementia, social cognition, theory of mind, emotional processing, social perception, attribution bias

In the published article, there was an error regarding the affiliation for author Rosa Ayesa-Arriola. As well as having affiliation 1, 2, they should also have the affiliation “5 CIBERSAM, Biomedical Research Network on Mental Health Area, Madrid, Spain.”

In the published article, there was an error in Table 7 as published. Table 7 was published without a row corresponding to Russell et al., 2020. The corrected Table 7 appears below.

**Table 7:** Summary of studies on social cognition in unspecific frontotemporal dementia.

**References**	**Design**	**FTD patients (*N*)**	**Mean age of FTD (S.D)**	**HC (N)**	**Mean age of HC (S.D)**	**SC domains**	**Assessment tools**	**Results**	**JBI**
Bedoin et al. (2009)	C-S	11	61.2 (3.2)	11	58.54 (5.3)	EP	*Ad hoc* test	Patients with impairment in EP	M
Zahn et al. (2009)	C-S	29	61.3 (8.9)	15	61.5 (8.5)	SP	*Ad hoc* test	Patients with impairment in SP	L
Bediou et al. (2009)	C-S	10	67.0 (7.0)	10	70.0 (6.0)	EP	*Ad hoc* test	FTD worse than AD	M
Formica et al. (2020)	C-S	14	74.29 (4.68)	N/A	N/A	ToM	RMET; SET	FTD worse than AD	M
Russell et al. (2020)	C-S	103	N/A	246	46 (12.8)	ToM; EP	FPT; FERT	FTD worse than controls	G

AD, Alzheimer Disease; CATS, Comprehensive Affect Testing System; C-S, Cross-sectional; E60, Ekman 60 task; EC, Ekman Caricatures; EM, Emotional Processing; FADT, Face Affect Discrimination Task; FAST, Facial Affect Selection test; FERT, Facial emotion recognition; FIDT, Face Identify Discrimination Task; FPT, Faux Pas test; FTD, Frontotemporal Dementia; G, Good; HC, Healthy Controls; IRI, Interpersonal Reactivity Index; JBI, Joanna Briggs Institute Critical Evaluation Checklists; L, Low; Lo, Longitudinal; LBD, Lewy Body Dementia; M, Moderate; mini-SEA, Mini-Social Cognition & Emotional Assessment; N/A, not available; nfvPPA, non-fluent variant PPA; PPA, Primary Progressive Aphasia; RMET, Reading the mind in the eyes test; SC, Social Cognition; SEA, Facial Emotion Recognition Test; SEQ, Socio-Emotional Questionnaire; SET, The Story-based Empathy Task; SP, Social Perception; svPPA, semantic variant PPA; TASIT, The Awareness of Social Inference Test; ToM, Theory of Mind.

The authors apologize for these errors and state that this does not change the scientific conclusions of the article in any way. The original article has been updated.

## Publisher's note

All claims expressed in this article are solely those of the authors and do not necessarily represent those of their affiliated organizations, or those of the publisher, the editors and the reviewers. Any product that may be evaluated in this article, or claim that may be made by its manufacturer, is not guaranteed or endorsed by the publisher.
