# Supplementary material for: Exploring the Relationship Between Deficits in Social Cognition and Neurodegenerative Dementia: A Systematic Review
Source: Front Aging Neurosci. 2022 Apr 27;14:778093. doi: 10.3389/fnagi.2022.778093 (PMC9093607; doi:10.3389/fnagi.2022.778093)
Supplement: Supplementary file 1 [file Table_1.docx]

| STUDY AUTHOR | Q1  Comparable groups | Q2  Groups matched appropriately | Q3  Same criteria for identification groups | Q4  Exposure measured in a standard, valid and reliable way | Q5  Exposure measured in the same way for groups | Q6  Confounding factors identified | Q7  Strategies to deal with confounding stated | Q8  Outcomes assessed in a standard, valid and reliable way | Q9  Exposure period enough | Q10  Appropriate statistical analysis |  |
| --- | --- | --- | --- | --- | --- | --- | --- | --- | --- | --- | --- |
| Bediou et al., 2009 | ✓ | ✓ | ✓ | ✓ | ✓ | 🗶 | 🗶 | 🗶 | N/A | ✓ | 66% |
| Bedoin et al., 2009 | ✓ | ✓ | ✓ | ✓ | ✓ | 🗶 | 🗶 | 🗶 | N/A | 🗶 | 55% |
| Fernandez-Duque et al., 2009 | ✓ | 🗶 | ✓ | ✓ | 🗶 | 🗶 | 🗶 | 🗶 | N/A | ✓ | 44% |
| Garcia-Rodriguez et al., 2009 | ✓ | ✓ | ✓ | ✓ | ✓ | 🗶 | 🗶 | ✓ | N/A | 🗶 | 66% |
| Guaita et al., 2009 | ✓ | 🗶 | ✓ | ✓ | ✓ | 🗶 | 🗶 | 🗶 | N/A | ✓ | 55% |
| Henry et al., 2009 | ✓ | ✓ | ✓ | ✓ | ✓ | 🗶 | 🗶 | ✓ | N/A | 🗶 | 66% |
| Kipps et al., 2009a | ✓ | 🗶 | ✓ | ✓ | ✓ | ✓ | ✓ | ✓ | N/A | ✓ | 88% |
| Kipps et al., 2009b | ✓ | 🗶 | ✓ | ✓ | ✓ | 🗶 | 🗶 | ✓ | N/A | ✓ | 66% |
| Rankin et al., 2009 | ✓ | 🗶 | ✓ | ✓ | ✓ | ✓ | ✓ | ✓ | N/A | ✓ | 88% |
| Torralva et al., 2009 | ✓ | ✓ | ✓ | ✓ | ✓ | 🗶 | 🗶 | ✓ | N/A | ✓ | 77% |
| Zahn et al., 2009 | ✓ | 🗶 | 🗶 | ✓ | 🗶 | ✓ | 🗶 | 🗶 | N/A | ✓ | 44% |
| Fernandez-Duque et al., 2010 | ✓ | 🗶 | ✓ | ✓ | ✓ | 🗶 | 🗶 | 🗶 | N/A | ✓ | 55% |
| Grossman et al., 2010 | ✓ | 🗶 | ✓ | ✓ | ✓ | 🗶 | 🗶 | 🗶 | N/A | ✓ | 55% |
| Youmans and Bourgeois 2010 | ✓ | ✓ | ✓ | ✓ | ✓ | 🗶 | 🗶 | ✓ | N/A | ✓ | 77% |
| Castelli et al., 2011 | ✓ | 🗶 | ✓ | ✓ | ✓ | 🗶 | 🗶 | ✓ | N/A | ✓ | 66% |
| Eslinger et al., 2011 | ✓ | ✓ | ✓ | ✓ | ✓ | 🗶 | 🗶 | ✓ | N/A | 🗶 | 66% |
| Kumfor et al., 2011 | ✓ | 🗶 | ✓ | ✓ | ✓ | 🗶 | 🗶 | ✓ | N/A | ✓ | 66% |
| Omar et al., 2011 | ✓ | 🗶 | 🗶 | ✓ | ✓ | ✓ | ✓ | ✓ | N/A | 🗶 | 66% |
| Cova et al., 2012 | ✓ | ✓ | ✓ | ✓ | ✓ | ✓ | ✓ | 🗶 | N/A | 🗶 | 77% |
| Goodkind et al., 2012 | ✓ | 🗶 | ✓ | ✓ | ✓ | ✓ | ✓ | 🗶 | N/A | ✓ | 77% |
| Le Bouc et al., 2012 | ✓ | ✓ | ✓ | ✓ | ✓ | ✓ | ✓ | ✓ | N/A | ✓ | 100% |
| Rohrer et al., 2012 | ✓ | ✓ | ✓ | ✓ | ✓ | 🗶 | 🗶 | ✓ | N/A | ✓ | 77% |
| Shany-Ur et al., 2012 | ✓ | ✓ | ✓ | ✓ | ✓ | ✓ | ✓ | ✓ | N/A | ✓ | 100% |
| Yamaguchi et al., 2012 | ✓ | 🗶 | ✓ | ✓ | ✓ | ✓ | ✓ | 🗶 | N/A | ✓ | 77% |
| Choong and Doody 2013 | ✓ | 🗶 | ✓ | ✓ | ✓ | 🗶 | N/A | ✓ | N/A | ✓ | 66% |
| Couto et al., 2013 | ✓ | ✓ | ✓ | 🗶 | ✓ | 🗶 | 🗶 | ✓ | N/A | ✓ | 66% |
| Freedman et al., 2013 | ✓ | 🗶 | ✓ | ✓ | ✓ | 🗶 | 🗶 | 🗶 | N/A | ✓ | 55% |
| Irish et al., 2013 | ✓ | 🗶 | ✓ | ✓ | ✓ | ✓ | ✓ | ✓ | N/A | ✓ | 88% |
| Laisney et al., 2013 | ✓ | ✓ | ✓ | ✓ | ✓ | 🗶 | 🗶 | ✓ | N/A | ✓ | 77% |
| Maki et al., 2013a | ✓ | 🗶 | ✓ | ✓ | ✓ | 🗶 | 🗶 | 🗶 | N/A | 🗶 | 44% |
| Maki et al., 2013b | ✓ | 🗶 | ✓ | ✓ | ✓ | ✓ | ✓ | 🗶 | N/A | 🗶 | 66% |
| Narme et al., 2013 | ✓ | 🗶 | ✓ | ✓ | ✓ | ✓ | ✓ | ✓ | N/A | ✓ | 88% |
| Savage et al., 2013 | ✓ | 🗶 | ✓ | ✓ | ✓ | ✓ | ✓ | ✓ | N/A | ✓ | 88% |
| Baez et al., 2014 | ✓ | ✓ | ✓ | ✓ | ✓ | ✓ | ✓ | ✓ | N/A | ✓ | 100% |
| Cerami et al., 2014 | ✓ | ✓ | ✓ | ✓ | ✓ | 🗶 | 🗶 | 🗶 | N/A | ✓ | 66% |
| Irish et al., 2014 | ✓ | 🗶 | ✓ | 🗶 | ✓ | 🗶 | 🗶 | 🗶 | N/A | ✓ | 44% |
| Kamminga et al., 2014 | ✓ | ✓ | ✓ | ✓ | ✓ | 🗶 | 🗶 | ✓ | N/A | ✓ | 77% |
| Kéri 2014 | ✓ | 🗶 | ✓ | ✓ | ✓ | 🗶 | 🗶 | ✓ | N/A | ✓ | 66% |
| Kumfor et al., 2014a | ✓ | 🗶 | ✓ | 🗶 | ✓ | 🗶 | 🗶 | ✓ | N/A | ✓ | 55% |
| Sollberger et al., 2014 | ✓ | 🗶 | ✓ | ✓ | ✓ | ✓ | ✓ | ✓ | N/A | 🗶 | 77% |
| Bertoux et al., 2015 | ✓ | 🗶 | ✓ | 🗶 | ✓ | 🗶 | 🗶 | ✓ | N/A | ✓ | 55% |
| Clark et al., 2015a | ✓ | 🗶 | ✓ | ✓ | 🗶 | 🗶 | 🗶 | ✓ | N/A | ✓ | 55% |
| Clark et al., 2015b | ✓ | 🗶 | ✓ | ✓ | ✓ | ✓ | ✓ | 🗶 | N/A | ✓ | 77% |
| Custodio et al., 2015 | ✓ | ✓ | ✓ | ✓ | ✓ | 🗶 | 🗶 | ✓ | N/A | ✓ | 77% |
| Downey et al., 2015 | ✓ | 🗶 | ✓ | ✓ | ✓ | ✓ | ✓ | ✓ | N/A | ✓ | 88% |
| El Haj et al., 2015 | ✓ | 🗶 | ✓ | ✓ | ✓ | 🗶 | 🗶 | ✓ | N/A | 🗶 | 55% |
| Hutchings et al., 2015 | ✓ | 🗶 | ✓ | ✓ | ✓ | ✓ | ✓ | ✓ | N/A | ✓ | 88% |
| Insch et al., 2015 | ✓ | ✓ | ✓ | ✓ | ✓ | 🗶 | 🗶 | 🗶 | N/A | 🗶 | 55% |
| Moyse et al., 2015 | ✓ | 🗶 | ✓ | ✓ | ✓ | 🗶 | 🗶 | 🗶 | N/A | 🗶 | 44% |
| Oliver et al. 2015 | ✓ | ✓ | ✓ | ✓ | ✓ | ✓ | ✓ | ✓ | N/A | ✓ | 100% |
| Torralva et al., 2015 | ✓ | ✓ | ✓ | ✓ | ✓ | 🗶 | 🗶 | ✓ | N/A | ✓ | 77% |
| Van den Stock et al., 2015 | ✓ | ✓ | ✓ | ✓ | ✓ | ✓ | ✓ | ✓ | N/A | ✓ | 100% |
| Baez et al., 2016 | ✓ | ✓ | ✓ | 🗶 | ✓ | ✓ | ✓ | 🗶 | N/A | ✓ | 77% |
| Bejanin et al., 2016 | ✓ | ✓ | ✓ | ✓ | ✓ | ✓ | ✓ | ✓ | N/A | ✓ | 100% |
| Binney et al., 2016 | ✓ | 🗶 | ✓ | ✓ | ✓ | ✓ | ✓ | ✓ | N/A | ✓ | 88% |
| Chiu et al., 2016 | ✓ | 🗶 | ✓ | ✓ | ✓ | ✓ | ✓ | ✓ | N/A | ✓ | 88% |
| Dermody et al., 2016 | ✓ | 🗶 | ✓ | ✓ | ✓ | ✓ | ✓ | 🗶 | N/A | ✓ | 77% |
| Fliss et al., 2016 | ✓ | 🗶 | ✓ | ✓ | ✓ | 🗶 | 🗶 | ✓ | N/A | ✓ | 66% |
| Heitz et al., 2016 | ✓ | 🗶 | ✓ | ✓ | ✓ | ✓ | ✓ | ✓ | N/A | ✓ | 88% |
| Jastorff et al., 2016 | ✓ | ✓ | ✓ | ✓ | ✓ | ✓ | ✓ | 🗶 | N/A | 🗶 | 77% |
| Moreau et al., 2016 | ✓ | 🗶 | ✓ | ✓ | ✓ | 🗶 | 🗶 | ✓ | N/A | ✓ | 66% |
| Sedeño et al., 2016 | ✓ | ✓ | ✓ | ✓ | ✓ | ✓ | ✓ | ✓ | N/A | ✓ | 100% |
| Tabernero and Politis 2016 | ✓ | ✓ | ✓ | ✓ | 🗶 | 🗶 | 🗶 | ✓ | N/A | ✓ | 66% |
| Fong et al., 2017a | ✓ | 🗶 | ✓ | ✓ | ✓ | 🗶 | 🗶 | 🗶 | N/A | ✓ | 55% |
| Fong et al., 2017 | ✓ | ✓ | ✓ | ✓ | ✓ | 🗶 | 🗶 | 🗶 | N/A | ✓ | 66% |
| Hazelton et al., 2017 | ✓ | 🗶 | ✓ | ✓ | ✓ | 🗶 | 🗶 | ✓ | N/A | ✓ | 66% |
| Insch et al., 2017 | ✓ | ✓ | ✓ | ✓ | 🗶 | 🗶 | 🗶 | 🗶 | N/A | ✓ | 55% |
| Kemp et al., 2017 | ✓ | ✓ | ✓ | ✓ | ✓ | 🗶 | 🗶 | ✓ | N/A | ✓ | 77% |
| Kumfor et al., 2017 | ✓ | 🗶 | ✓ | 🗶 | ✓ | ✓ | ✓ | ✓ | N/A | ✓ | 77% |
| Multani et al., 2017 | ✓ | 🗶 | ✓ | ✓ | ✓ | ✓ | ✓ | ✓ | N/A | ✓ | 88% |
| Park et al., 2017 | 🗶 | 🗶 | ✓ | ✓ | ✓ | ✓ | ✓ | ✓ | N/A | ✓ | 77% |
| Poveda et al., 2017 | ✓ | 🗶 | ✓ | ✓ | ✓ | 🗶 | 🗶 | ✓ | N/A | 🗶 | 55% |
| Ramanan et al., 2017 | ✓ | 🗶 | ✓ | ✓ | ✓ | 🗶 | 🗶 | ✓ | N/A | ✓ | 66% |
| Reul et al., 2017 | ✓ | 🗶 | ✓ | ✓ | ✓ | ✓ | ✓ | ✓ | N/A | ✓ | 88% |
| Sava et al., 2017 | ✓ | ✓ | ✓ | ✓ | ✓ | 🗶 | 🗶 | 🗶 | N/A | ✓ | 66% |
| Santamaría-García et al., 2017 | ✓ | ✓ | ✓ | ✓ | 🗶 | ✓ | ✓ | ✓ | N/A | ✓ | 88% |
| Simm et al., 2017 | 🗶 | 🗶 | ✓ | ✓ | ✓ | 🗶 | 🗶 | ✓ | N/A | ✓ | 55% |
| Sturm et al., 2017 | ✓ | ✓ | ✓ | ✓ | ✓ | ✓ | ✓ | 🗶 | N/A | 🗶 | 77% |
| Tabernero et al., 2017 | ✓ | 🗶 | 🗶 | ✓ | ✓ | 🗶 | 🗶 | ✓ | N/A | ✓ | 55% |
| Van den Stock et al., 2017 | ✓ | 🗶 | ✓ | ✓ | ✓ | ✓ | ✓ | ✓ | N/A | 🗶 | 77% |
| Wong et al., 2017 | ✓ | 🗶 | ✓ | ✓ | ✓ | 🗶 | 🗶 | 🗶 | N/A | ✓ | 55% |
| Zahn et al., 2017 | ✓ | ✓ | ✓ | ✓ | ✓ | ✓ | ✓ | ✓ | N/A | ✓ | 100% |
| Carr et al., 2018 | ✓ | ✓ | ✓ | ✓ | ✓ | 🗶 | 🗶 | ✓ | N/A | ✓ | 77% |
| Chen et al., 2018 | ✓ | ✓ | ✓ | ✓ | ✓ | 🗶 | 🗶 | ✓ | N/A | ✓ | 77% |
| Daley et al., 2018 | ✓ | 🗶 | 🗶 | ✓ | ✓ | 🗶 | 🗶 | ✓ | N/A | ✓ | 55% |
| Duclos et al., 2018 | ✓ | ✓ | ✓ | ✓ | ✓ | ✓ | ✓ | ✓ | N/A | ✓ | 100% |
| Kumfor et al., 2018a | ✓ | 🗶 | ✓ | 🗶 | ✓ | 🗶 | 🗶 | 🗶 | N/A | ✓ | 44% |
| Kumfor et al., 2018b | ✓ | 🗶 | ✓ | ✓ | ✓ | 🗶 | 🗶 | ✓ | N/A | ✓ | 66% |
| Marshall et al., 2018 | ✓ | 🗶 | ✓ | ✓ | ✓ | 🗶 | 🗶 | 🗶 | N/A | ✓ | 55% |
| Perri et al., 2018 | ✓ | ✓ | 🗶 | ✓ | ✓ | 🗶 | 🗶 | 🗶 | N/A | ✓ | 55% |
| Sava et al., 2018 | ✓ | ✓ | ✓ | ✓ | ✓ | 🗶 | 🗶 | 🗶 | N/A | ✓ | 66% |
| Schroeter et al., 2018 | ✓ | ✓ | ✓ | ✓ | ✓ | 🗶 | 🗶 | ✓ | N/A | ✓ | 77% |
| Sturm et al., 2018 | ✓ | 🗶 | ✓ | ✓ | ✓ | ✓ | ✓ | 🗶 | N/A | ✓ | 77% |
| Synn et al., 2018 | ✓ | ✓ | ✓ | ✓ | ✓ | ✓ | ✓ | ✓ | N/A | ✓ | 100% |
| Takenoshita et al., 2018 | ✓ | 🗶 | ✓ | ✓ | ✓ | 🗶 | 🗶 | ✓ | N/A | ✓ | 66% |
| Yamaguchi et al., 2018 | ✓ | 🗶 | ✓ | ✓ | ✓ | ✓ | ✓ | 🗶 | N/A | ✓ | 77% |
| García-Casal et al., 2019 | 🗶 | ✓ | ✓ | ✓ | ✓ | ✓ | ✓ | ✓ | N/A | ✓ | 88% |
| Giovagnoli et al., 2019 | ✓ | ✓ | ✓ | ✓ | ✓ | 🗶 | 🗶 | ✓ | N/A | ✓ | 77% |
| Lozachmeur et al., 2019 | ✓ | 🗶 | ✓ | ✓ | ✓ | 🗶 | 🗶 | ✓ | N/A | 🗶 | 55% |
| Van de Stock et al., 2019 | ✓ | ✓ | ✓ | ✓ | ✓ | ✓ | ✓ | ✓ | N/A | ✓ | 100% |
| Arroyo-Anlló et al. 2019 | ✓ | 🗶 | ✓ | ✓ | ✓ | 🗶 | 🗶 | ✓ | N/A | ✓ | 66% |
| El Haj et al. 2019 | ✓ | ✓ | ✓ | ✓ | ✓ | 🗶 | 🗶 | ✓ | N/A | ✓ | 77% |
| Chainay et al. 2020 | ✓ | 🗶 | ✓ | ✓ | ✓ | 🗶 | 🗶 | ✓ | N/A | ✓ | 66% |
| Kawano et al., 2020 | ✓ | 🗶 | ✓ | ✓ | ✓ | 🗶 | 🗶 | ✓ | N/A | ✓ | 66% |
| Lillo et al., 2020 | ✓ | 🗶 | ✓ | ✓ | ✓ | 🗶 | 🗶 | ✓ | N/A | ✓ | 66% |
| Bertoux et al., 2020 | ✓ | 🗶 | 🗶 | ✓ | 🗶 | 🗶 | 🗶 | ✓ | N/A | ✓ | 44% |
| Russell et al., 2020 | ✓ | ✓ | ✓ | ✓ | ✓ | 🗶 | 🗶 | ✓ | N/A | ✓ | 77% |
| Hayashi and Terada 2021 | ✓ | 🗶 | ✓ | ✓ | ✓ | 🗶 | 🗶 | ✓ | N/A | ✓ | 66% |
