# Supplementary material for: Exploring the Relationship Between Deficits in Social Cognition and Neurodegenerative Dementia: A Systematic Review
Source: Front Aging Neurosci. 2022 Apr 27;14:778093. doi: 10.3389/fnagi.2022.778093 (PMC9093607; doi:10.3389/fnagi.2022.778093)
Supplement: Supplementary file 2 [file Table_2.docx]

| STUDY AUTHOR | Q1  Clearly defined inclusion criteria | Q2  Subjects and the setting described in detail | Q3  Exposure measured in a valid and reliable way | Q4  Objective, standard criteria for measurement | Q5  Confounding criteria identified | Q6  Strategies to deal with confounding stated | Q7  Outcomes measured in a valid and reliable way | Q8  Appropriate statistical analysis |  |
| --- | --- | --- | --- | --- | --- | --- | --- | --- | --- |
| Buhl et al., 2013 | ✓ | ✓ | ✓ | ✓ | ✓ | ✓ | ✓ | ✓ | 100% |
| Hsieh et al., 2013 | ✓ | ✓ | ✓ | 🗶 | 🗶 | 🗶 | 🗶 | ✓ | 50% |
| Miller et al., 2013 | ✓ | ✓ | ✓ | ✓ | 🗶 | 🗶 | ✓ | ✓ | 75% |
| Cerami et al., 2015 | ✓ | ✓ | ✓ | ✓ | 🗶 | 🗶 | ✓ | ✓ | 75% |
| Carr et al., 2017 | ✓ | ✓ | ✓ | ✓ | ✓ | ✓ | ✓ | ✓ | 100% |
| Dodich et al., 2018 | ✓ | ✓ | ✓ | ✓ | 🗶 | 🗶 | ✓ | ✓ | 75% |
| Dourado et al. 2019 | ✓ | ✓ | ✓ | ✓ | ✓ | ✓ | ✓ | ✓ | 100% |
| Formica et al., 2020 | ✓ | ✓ | ✓ | 🗶 | 🗶 | 🗶 | ✓ | ✓ | 62% |
