# Supplementary material for: Exploring the Relationship Between Deficits in Social Cognition and Neurodegenerative Dementia: A Systematic Review
Source: Front Aging Neurosci. 2022 Apr 27;14:778093. doi: 10.3389/fnagi.2022.778093 (PMC9093607; doi:10.3389/fnagi.2022.778093)
Supplement: Supplementary file 3 [file Table_3.docx]

| STUDY AUTHOR | Q1  Groups similar from same population | Q2  Exposures measured similarly | Q3  Exposure measured in a valid and reliable way | Q4  Confounding factors identified | Q5  Strategies to deal with confounding stated? | Q6  Participants free of outcome | Q7  Outcomes measured in a valid and reliable way | Q8  Appropriate follow-up time | Q9  Complete follow-up | Q10  Incomplete follow-up strategies used | Q11  Appropriate statistical analysis |  |
| --- | --- | --- | --- | --- | --- | --- | --- | --- | --- | --- | --- | --- |
| Kumfor et al., 2014 | ✓ | ✓ | ✓ | 🗶 | 🗶 | N/A | ✓ | ✓ | 🗶 | 🗶 | ✓ | 60% |
| Torres et al., 2015 | ✓ | ✓ | 🗶 | 🗶 | 🗶 | ✓ | 🗶 | ✓ | ✓ | N/A | ✓ | 60% |
| Kumfor et al., 2016 | ✓ | ✓ | ✓ | 🗶 | 🗶 | N/A | ✓ | ✓ | 🗶 | 🗶 | ✓ | 60% |
| Garcia-Casal et al., 2017 | ✓ | ✓ | ✓ | 🗶 | 🗶 | N/A | ✓ | ✓ | ✓ | 🗶 | ✓ | 70% |
| Gossink et al., 2018 | ✓ | ✓ | ✓ | 🗶 | 🗶 | ✓ | ✓ | ✓ | 🗶 | 🗶 | ✓ | 63% |
| Reus et al., 2018 | ✓ | ✓ | ✓ | 🗶 | 🗶 | ✓ | ✓ | ✓ | ✓ | ✓ | ✓ | 82% |
