# Supplementary material for: Exploring the Relationship Between Deficits in Social Cognition and Neurodegenerative Dementia: A Systematic Review
Source: Front Aging Neurosci. 2022 Apr 27;14:778093. doi: 10.3389/fnagi.2022.778093 (PMC9093607; doi:10.3389/fnagi.2022.778093)
Supplement: Supplementary file 4 [file Data_Sheet_1.docx]

Search strings MEDLINE (Pubmed), Cochrane Library, Lilacs, Web of Science (WoS) and PsycINFO

**Pubmed**

"Dementia"[Mesh] AND ("Social Cognition" OR "Theory of Mind"[Mesh] OR "Social Perception"[Mesh] OR "Emotional Intelligence"[Mesh] OR "Social Knowledge" OR "Attributional Style" OR "Attributional Bias") AND "last 10 years"[PDat])

**Cochrane Library**

**
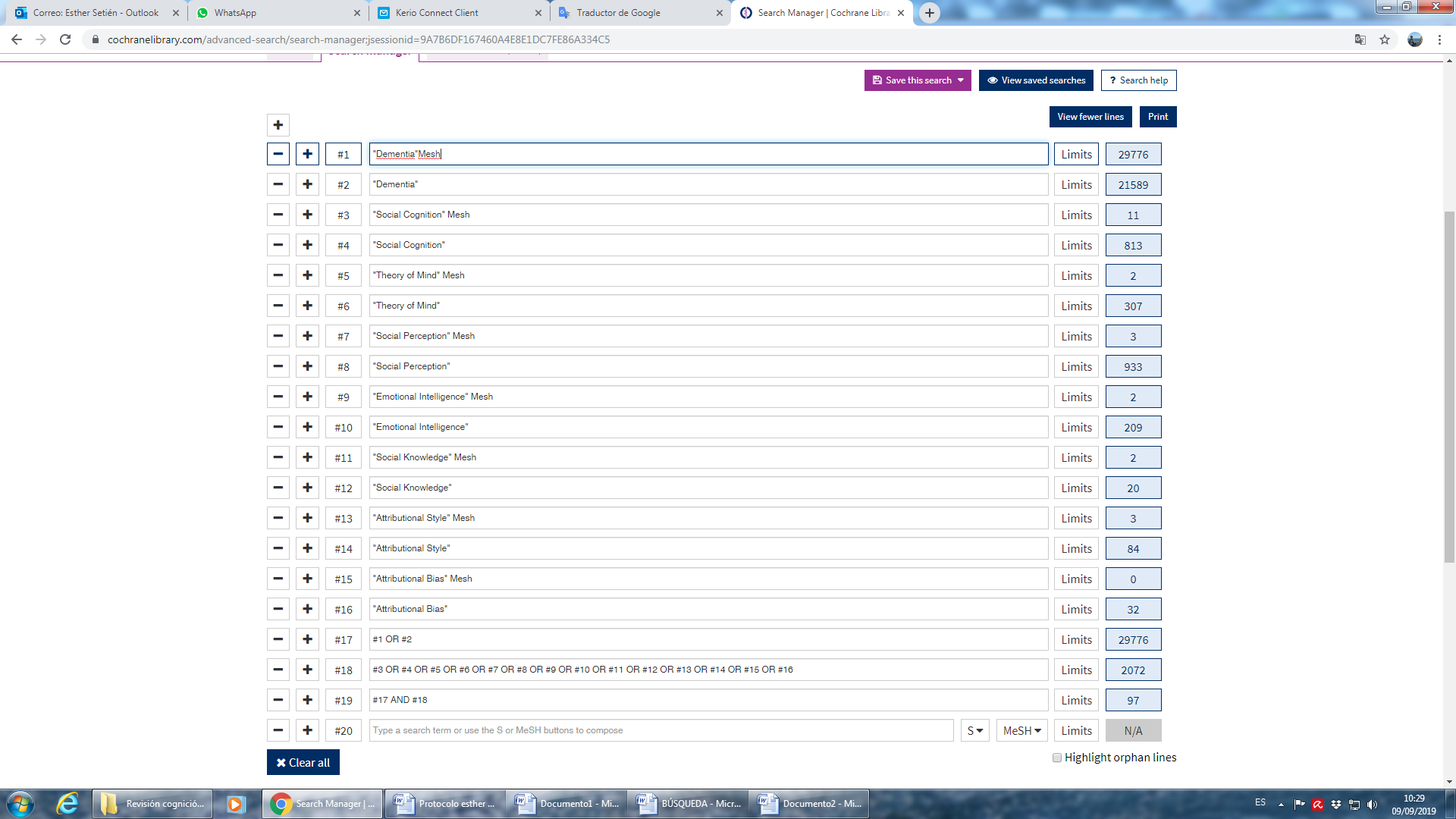
**

**Lilacs**

tw:"dementia" AND (tw:"Social Cognition" OR tw:"Theory of Mind" OR tw:"Social Perception" OR tw:"Emotional Intelligence" OR tw:"Social Knowledge" OR tw:"Attributional Style" OR tw:"Attributional Bias")

**Web of Science (WoS)**

TS="dementia" AND TS=("Social Cognition" OR "Theory of Mind" OR "Social Perception" OR "Emotional Intelligence" OR "Social Knowledge" OR "Attributional Style" OR "Attributional Bias")

**PsycINFO:**

(DE "Dementia") AND ((DE "Social Cognition") OR (DE "Theory of Mind") OR (DE "Social Perception") OR (DE "Emotional Intelligence") OR ("Social Knowledge") OR ("Attributional Style") OR (Attributional Bias")).
